# Structure and Carbon Dioxide Adsorption Properties of a Nanosized Aluminum l‑Aspartate Metal–Organic Framework

**DOI:** 10.1021/acsanm.6c00412

**Published:** 2026-04-21

**Authors:** Letizia Trovarelli, Virginia Guiotto, Maria Sole Notari, Lorenzo Isidoro, Giacomo Provinciali, Concetta Bafaro, Andrea Rossin, Martino Degli Innocenti, Naomi Anna Consoli, Moreno Lelli, Marco Taddei, Matteo Signorile, Valentina Crocellà, Ferdinando Costantino

**Affiliations:** † Dipartimento di Chimica, Biologia e Biotecnologie (DCBB), 201791 Università di Perugia, Via Elce di Sotto 8, 06123 Perugia,Italy; ‡ Dipartimento di Chimica, Centro di Riferimento NIS, Unità di Ricerca INSTM, 9314 Università degli Studi di Torino, Via G. Quarello 15/A and Via P. Giuria 7, I-10125 Torino, Italy; § Istituto di Chimica dei Composti Organometallici (CNR-ICCOM), Via Madonna del Piano 10, Sesto Fiorentino (Firenze) 50019, Italy; ∥ Centre of Magnetic Resonance (CERM) Università di Firenze, Via Luigi Sacconi 6, Sesto Fiorentino (Firenze) 50019, Italy; ⊥ Dipartimento di Chimica Ugo Schiff, 9300 Università di Firenze, Via della Lastruccia 3-13, Sesto Fiorentino (Firenze) 50019, Italy; # Dipartimento di Chimica e Chimica Industriale, Unità di Ricerca INSTM, 9310 Università di Pisa, Via Giuseppe Moruzzi 13, 56124 Pisa, Italy

**Keywords:** metal−organic frameworks, carbon dioxide adsorption, FT-IR spectroscopy, ss-NMR spectroscopy, DFT calculations

## Abstract

Herein, we report on the green synthesis of a nanosized (40 nm average size platelet crystals) aluminum-based metal–organic framework (MOF) [Al­(OH)­(l-Asp)­(HNO_3_)_0.31_]·1.5H_2_O (l-H_2_Asp = l-aspartic acid; **Al-l-Asp**), composed of nanometric crystals and isostructural to Al-fumarate. The synthesis was performed under mild conditions using γ-valerolactone (GVL), a solvent derived from biomass valorization. The MOF structure, modeled through DFT calculations, is constituted of 1D-infinite Al-hydroxo chains connected by the carboxylic groups of l-aspartic acid with the same framework topology as that of Al-MIL-53. **Al-l-Asp** displays thermal stability up to 240 °C, and no breathing effects are observed upon thermal activation. Solid-state NMR was used to investigate the activation process and chemical details of the pore structure and activation dynamics. The activated MOF has been exploited in carbon dioxide adsorption. The BET surface area is about 600 m^2^·g^–1^, and the MOF CO_2_ capture capacity reaches 3.2 mmol·g^–1^ at *T* = 0 °C and *p*_CO_2__ = 1 bar. Remarkably, compared to the Al-fumarate analogue, the CO_2_ heat of adsorption is increased from about 20 kJ·mol^–1^ to a value of 30 kJ·mol^–1^, proving the beneficial effect of the presence of an amino group on the linker skeleton on the CO_2_-MOF interaction. CO_2_/N_2_ IAST selectivity is relatively high, ranging from 60 to more than 80 at increasing pressure range (from 0.1 to 1 bar). These values overcome those measured for the analogue fumarate MOF, and they are comparable to the benchmark compounds for CO_2_ in postcombustion adsorption applications.

## Graphical abstract

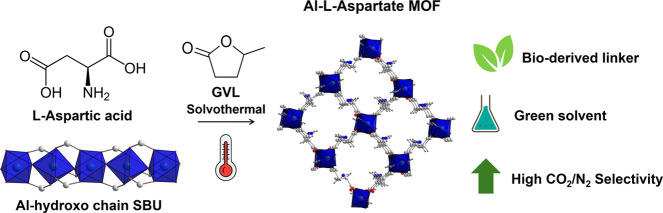

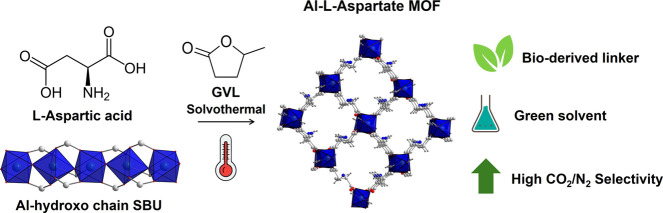

## Introduction

The greenhouse effect is a fundamental physical process by which certain gases in Earth’s atmosphere trap heat radiating from the planet’s surface, thereby warming the lower atmosphere and surface. Without this natural effect, Earth’s average temperature would be about 33 °C cooler, making the planet largely uninhabitable. However, human activities (especially the burning of fossil fuels, such as coal, oil, and gas) have significantly increased the concentrations of greenhouse gases, notably carbon dioxide (CO_2_), methane (CH_4_), and nitrous oxide (N_2_O). These gases enhance the natural greenhouse effect, driving global warming and broader climate change. Among these gases, CO_2_ is the most abundant and persistent long-lived greenhouse gas emitted by human actions. Observational records show that atmospheric CO_2_ concentrations have risen from about 278 ppmin the preindustrial era (around 1750) to over 420 ppm nowadays, representing more than a 50% increase compared with preindustrial levels. These elevated CO_2_ levels are now locking in long-term warming due to their long atmospheric lifetime and continued emissions from energy production, transportation, industry, and land-use changes. In this context, metal–organic frameworks (MOFs) have emerged as highly promising materials for mitigating CO_2_ emissions and addressing climate change. MOFs are crystalline porous solids composed of metal nodes and organic linkers characterized by exceptionally high surface areas, tunable pore sizes, and adjustable chemical functionalities. These features make them particularly attractive for CO_2_ capture, separation, and storage, including postcombustion capture, direct air capture, and selective gas separation processes.^1,2^ Ongoing research has demonstrated that the rational design of MOF structures can significantly enhance the CO_2_ adsorption capacity, selectivity, and regeneration efficiency, positioning MOFs as key candidates for next-generation carbon management technologies. Among the countless MOF families, aluminum-based MOFs (Al-MOFs) represent an important class of porous materials distinguished not only by their high thermal, chemical, and hydrolytic stability but also by the low atomic weight of aluminum, which contributes to a reduced framework density and a high gravimetric adsorption efficiency.^3–5^ Built from light, abundant, and nontoxic Al­(III) centers, Al-MOFs typically form robust inorganic building units connected by carboxylate linkers, yielding frameworks that remain stable under harsh conditions, including moisture and acidic gases. One of the most famous aluminum MOFs, which is currently produced at an industrial scale, is Al-fumarate (Al-FUM), marketed under the commercial name of Basolite A520 by the BASF Chemical Company.^6^ Al-FUM, [Al­(OH)­(FUM)·4H_2_O] (FUM^2–^ = fumarate), is isostructural with the terephthalate analogue Al-MIL-53 MOF, but with a rigid structure and smaller pores. Al-FUM has been exploited in many applications ranging from gas storage and separation,^7–9^ pollution remediation,^10^ heterogeneous catalysis,^11^ and as a filler for mixed-matrix membranes.^12^

A key strategy for enhancing the sustainability of Al-MOFs is the use of natural molecules as organic linkers, such as amino acids, sugars, or polyphenols. These bioderived linkers are renewable, biodegradable, and often nontoxic, reducing the environmental footprint of MOF production. The combination of light framework weight, structural robustness, and green linker chemistry makes Al-MOFs particularly attractive for CO_2_ capture and separation, gas storage, and other adsorption-based applications where both performance and sustainability are critical. Such bioinspired MOFs find applications as biomaterials,^13^ biosensors,^14^ asymmetric heterogeneous catalysis,^15^ for proton conductivity^16^ and gas separation.^17^ Among natural amino acids, aspartate is an α-amino acid that can also be described as a C_4_ analogue of succinic acid containing an amino group linked to the α-chiral carbon atom of the chain. α-d-Aspartate is essential in many biochemical processes, such as protein synthesis, and treatment of diseases, like male infertility, neuropsychiatry, and blood detoxification.^18^ Aspartic acid, in its pure enantiomeric forms or as d,l-racemate, was recently employed for the synthesis of several MOFs based on high valent (Ce, Zr),^19,20^ alkaline (Na, Ca),^21,22^ or transition metals (Zn, Ni, Cu).^23–28^

Inspired by the possibility to expand the family of bio-Al-MOFs for carbon capture applications, in this work, we report on the synthesis and thorough solid-state characterization, based on PXRD, FT-IR, and ss-NMR, of a novel aluminum-based l-aspartate MOF (hereafter **Al-l-Asp**) of minimal formula [Al­(OH)­(l-Asp)­(HNO_3_)_0_._3_·1.5H_2_O] isostructural to Al-FUM. The MOF, characterized by nanometric crystals, was prepared using γ-valerolactone (GVL) as solvent, following the principles of a more sustainable synthetic chemistry. Finally, its CO_2_ adsorption properties were evaluated at three different temperatures, resulting in good adsorption capacity and IAST CO_2_/N_2_ adsorption selectivity, recording values comparable to many benchmark compounds for CO_2_ adsorption application in postcombustion gas upgrade.

## Materials and Methods

### Synthesis of Al-l-Aspartate MOF (**Al-l-Asp**)

A mixture of 1875 mg (5 mmol) of aluminum nitrate nonahydrate [Al­(NO_3_)_3_·9H_2_O] (Sigma-Aldrich), and 665 mg (5 mmol) of l-aspartic acid (Sigma-Aldrich) was dissolved in 5 mL (50 mmol) of GVL (Sigma-Aldrich) and placed in a poly­(tetrafluoroethylene) (PTFE, Teflon)-lined hydrothermal reactor. The sealed reactor was heated in a static oven at 120 °C for 24 h. The resulting white solid was washed twice with 20 mL of hot (70 °C) deionized water for 1 h each time. The purified product was then dried overnight in a static oven at 60 °C. Yield: 900 mg (80%), based on the formula [Al­(OH)­(l-Asp)­(HNO_3_)_0.31_] ICP-OES analysis: Al wt % obs 15.5%, calcd 13.6%.

### Synthesis of Al-Fumarate (Basolite A520)

The synthesis of Al-fumarate was carried out following the procedure reported in the literature patent.^6^ 70 g (0.105 mol) of aluminum sulfate hydrate [Al_2_(SO_4_)_3_·18H_2_O] (Sigma-Aldrich) was dissolved in 300 g of water (16.6 mol) and heated to 60 °C. To this solution, a mixture containing 24.47 g (0.211 mol) of fumaric acid (Sigma-Aldrich), 25.32 g (0.633 mol) of NaOH and 661.7 g (36.8 mol) of water was added over 1 h of total mixing time. During the addition, the white powder of Al-fumarate precipitates out of the reaction mixture. The obtained solid product was washed 3 times with 500 mL of deionized water. The purified product was then dried overnight in a static oven at 60 °C. Yield: 76% based on the formula [Al­(OH)­(FUM)]·4H_2_O.

### In Situ FT-IR Spectroscopy

FT-IR measurements were performed within the 4000–500 cm^–1^ spectral range using a Bruker Vertex 70 spectrophotometer equipped with an MCT (mercury cadmium tellurium) cryogenic detector. The resolution of the reported IR spectra is 2.0 cm^–1^, and an average of 32 scans was used to increase the signal-to-noise ratio. Before the analysis, the sample, in the form of a self-supported pellet mechanically protected by a gold envelope, was inserted into a homemade quartz cell with KBr windows. To evaluate the optimal activation conditions, multiple spectra were acquired during in situ degassing at room temperature (RT) by using a high-vacuum glass line, equipped with mechanical and turbomolecular pumps (residual pressure *p* < 10^–4^ mbar). Finally, an additional ex situ treatment at 200 °C (3 °C·min^–1^) overnight was necessary to completely activate the sample.

### Gas Sorption Analysis

Adsorption/desorption isotherms with different gases were collected by using a Micromeritics 3FLEX sorption analyzer. For measurements with N_2_ at −196 °C, the cooling bath was prepared using liquid nitrogen. Before analysis, the materials were treated by coupling dynamic vacuum and a temperature program according to the required activation conditions (150° and 200 °C overnight for **Al-l-Asp**; 100 °C for Al-FUM).

Brunauer–Emmet–Teller (BET) surface area was evaluated in the 5 × 10^–3^ to 4 × 10^–2^ *p*/*p*_o_ range, according to the Rouquerol consistency criteria.^29^

The analysis of the pore size distribution (PSD) and cumulative pore volume (CPV) was performed by applying the nonlocal density functional theory (NL-DFT) method by using the MicroActive Software provided by Micromeritics. For each N_2_ isotherm, the kernel of the isotherms with the best fit was selected. For both MOFs a cylindrical pore geometry was selected, and the isotherms were fitted with the models “N_2_@77-Oxide Cylindrical Pores, Tarazona” (**Al-l-Asp**) and “N_2_@77 Cyl Pores in Pillared Clay, NLDFT” (Al-FUM). The textural properties of **Al-l-Asp** were also evaluated theoretically using the software Zeo++.^30^ The accessible surface area (ASA) and the probe-occupiable volume were evaluated by using a pore diameter of 2.8 Å.

CO_2_ and N_2_ isotherms collected in the temperature range of 0–20 °C were performed using a chiller dewar from Micromeritics, in which a coolant or heating fluid, connected to a thermostatic bath (JULABO F25), can recirculate. The isosteric heat of adsorption was calculated by collecting three isotherms at different temperatures and applying the Clausius–Clapeyron equation.

$$\ln\frac{p_{1}}{p_{2}}=Q_{\mathrm{st}}\times \frac{T_{2}-T_{1}}{R\times T_{1}\times T_{2}}$$ 1

CO_2_/N_2_ selectivity was predicted starting from the experimental isotherms collected at 0 °C for each probe gas using the ideal adsorbed solution theory (IAST), as implemented in the software IAST++.^31^

### Powder X-Ray Diffraction (PXRD)

PXRD patterns were collected with a PANalytical X’pert PRO diffractometer equipped with a Cu Kα_1,2_ anode (λ = 1.5406 Å) operating at 40 kV and 40 mA. Data were collected in a Bragg–Brentano geometry, over a 2θ range of 5–40° for the control patterns and to 80° of 2θ for Pawley refinement. X’Pert HighScore Plus and Mercury software were used for data visualization.

### Variable-Temperature Powder X-Ray Diffraction (VT-PXRD)

VT-PXRD patterns were collected in the 5–40° 2θ range using a step size of 0.03° 2θ and a scanning rate of 10° 2θ min^–1^ with a Rigaku MiniFlex 600-C diffractometer working in Bragg–Brentano geometry and equipped with a D/teX detector, using Cu Kα radiation (λ = 1.5406 Å). The X-ray tube was operated at a voltage of 40 kV and a current of 15 mA. An Anton Paar BTS-500 chamber was employed to control the temperature of the sample.

### Scanning Electron Microscopy (SEM)

SEM images were obtained using an LEO 1525 Gemini SEM (Zeiss/LEO) with an acceleration voltage of 5.00 kV (USA). The sample was sputter-coated under a vacuum with chromium.

### Thermogravimetric Analysis (TGA)

TGA measurements were performed using a NETZSCH STA 2500 Regulus analyzer. Approximately 10 mg of sample was placed in a platinum pan for STA2500 and heated from 13 to 1110 °C at a heating rate of 10 °C·min^–1^ under a continuous flow of air atmosphere (N_2_/O_2_ = 70:30). The analysis was carried out to evaluate the thermal stability and decomposition behavior. TGAs combined with mass spectrometry for mass analysis of the volatile species were performed under a N_2_ flow (100 mL·min^–1^) at a heating rate of 5 °C·min^–1^ with an EXSTAR Thermo Gravimetric Analyzer Seiko 6200 coupled with a ThermoStar GSD 301T mass spectrometer.

### DFT Calculations

DFT calculations were performed with the CRYSTAL17 code,^32^ by adopting the B3LYP functional^33,34^ in conjunction with the Ahlrichs def2-SVP for all elements.^35^ Dispersive forces were included through the Grimme’s D3 scheme^36^ with Becke–Johnson damping.^36^ *k*-Space was sampled with a Pack–Monkhorst grid including 8 *k*-points. All other parameters defaulted to standard settings as defined in the CRYSTAL17 manual.^37^ The structural model of **Al-l-Asp** was obtained from the Al-FUM framework^38^ by modifying the organic linker from fumaric to l-aspartic acid and imposing cell parameters and space group as from Pawley refinement of the PXRD data. This structure was optimized (atomic positions only), and vibrational frequencies were simulated on the relaxed model. IR intensities were computed, as well.

### Multinuclear Solid-State NMR Spectroscopy (ss-NMR)

1D and 2D solid-state NMR spectra were acquired on two spectrometers: a Bruker Avance III spectrometer working at 20.0 T (corresponding to 850 MHz for ^1^H, 222 MHz for ^27^Al, 214 MHz for ^13^C, 86 MHz for ^15^N nuclear Larmor frequencies) and a Bruker NEO spectrometer working at 16.4 T (corresponding to 701 MHz for ^1^H, 183 MHz for ^27^Al, 176 MHz for ^13^C nuclear Larmor Frequencies) both operating with a 3.2 mm triple-resonance MAS probe (^1^H/^13^C/^15^N) at 20 kHz of MAS (unless otherwise specified in the figures caption) at a probe temperature incoming air of −3 °C. The reported ^1^H, ^13^C, ^27^Al, ^15^N chemical shifts are referenced to external standards: Si­(CH_3_)_4_, (TMS, ^1^H, ^13^C), Al­(NO_3_)_3_ in D_2_O for aluminum-27, liquid NH_3_, for nitrogen-15. In all the {^1^H}-X CP-MAS experiments 1H power was linearly ramped from 70 to 100%, while the X heteronuclei were irradiated constant power with a specific contact time indicated in the figure captions. During acquisition, ^1^H decoupling was performed by using SPINAL64. All 2D ^1^H–X, (X = ^13^C or^15^N), HETCOR (Heteronuclear Correlation) spectra were acquired by applying a frequency switched Lee–Goldburg cross polarization homonuclear correlation (FSLG) {^1^H–^1^H} decoupling in the indirect dimension. Detailed decoupling and CP powers and all the acquisition parameters are listed in the Supporting Information (Tables S14–S49).

## Results and Discussion

### Synthesis and Preliminary Characterization

The synthesis of **Al-l-Asp** was carried out by using GVL, a green solvent derived from biomasses conversion and used for several applications (Scheme 1).^39^ GVL is considered a more sustainable alternative to widely used organic solvents such as *N*-methyl-2-pyrrolidone (NMP) and *N*,*N*-dimethylformamide (DMF). It retains the essential characteristics of an effective solvent, including a high boiling point (207 °C) and a low melting point (−31 °C), making it liquid at room temperature. Like DMF, GVL is a polar, aprotic solvent that is thermally stable, showing no decomposition up to 150 °C in the absence of water. GVL can be readily derived from cellulose or hemicellulose, making it accessible from nonfood lignocellulosic biomass. In addition to its use as a solvent, GVL has applications in the pharmaceutical, fragrance, and food industries due to its mild, pleasant odor. A comparison with commonly used polar organic solvents shows that GVL is significantly less hazardous to human health than solvents such as NMP and DMF, while offering comparable or even lower levels of ecotoxicity. Its renewable origin, low toxicity to humans and the environment, and favorable physicochemical properties make GVL a highly promising candidate as a sustainable, green solvent.^40–42^ The use of GVL for the synthesis of a Zr-MOF has already been reported by our group.^41^

**Scheme 1:**
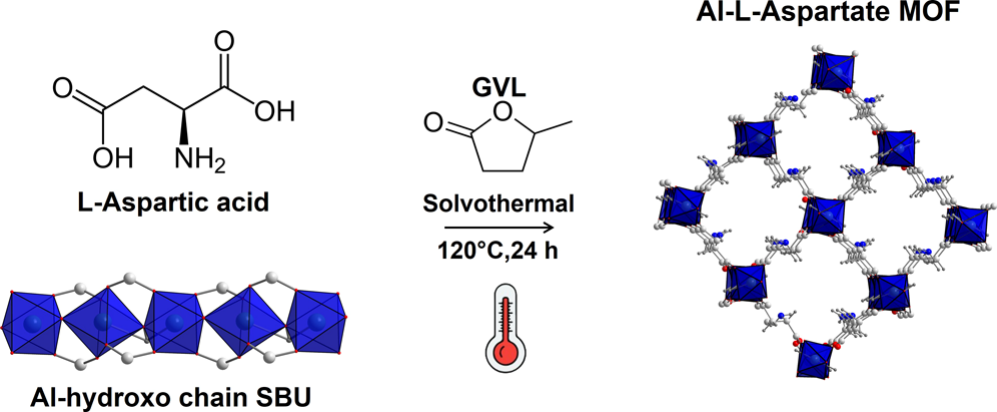
Solvothermal Synthesis of **Al-l-Asp** MOF and Representation of Its Crystal Structure

Attempts to obtain the MOF in other solvents (alcohols, DMF, and water) failed, whereas the only use of GVL led to the more crystalline product, as shown in Figure S1. Notably, the synthesis has been easily performed up to the gram scale (900 mg). The removal of GVL from the pores is essential to ensure efficient activation. For that reason, an intensive workup treatment in hot water (70 °C) was carried out. Hereafter, we will refer to the material after the water workup as **Al-l-Asp_ww** (where ww stands for the water workup). TGA was performed on the sample after being stirred in hot water (Figure S2). The TGA curve shows a first weight loss of about 10%, at 100 °C, attributed to the removal of water molecules. Then, a plateau is observed up to 220 °C, after which a second steep weight loss of 20% is present; however, this loss cannot be related to MOF decomposition, as confirmed by VT-PXRD measurements. Indeed, as observed in Figure S3, **Al-l-Asp_ww** retains its crystallinity up to approximately 240 °C, as evidenced by the presence of the MOF reflections throughout the heating process. The diffraction peaks remained largely unchanged in position up to 220 °C, indicating that the framework maintains its long-range order. Upon further heating, gradual peak broadening was observed, suggesting partial framework decomposition. The reflections around 32° of 2θ gradually disappeared as the temperature rose, in accordance with solvent evaporation, as shown in Figure S7. The reflections at around 34.4° and 37.0°, indexed as the (104) and (110) *hkl* planes of Al_2_O_3_, can be attributed to the alumina sample holder.

SEM analysis (Figure S4) shows that the **Al-l-Asp_ww** nanocrystals are around 40 nm in size. The MOF exhibits a flower-like morphology, formed by aggregated nanosheets or plate-like crystallites. These structures have a rough surface texture, indicating oriented growth.

### DFT Calculations and Structural Description

The experimental PXRD pattern of **Al-l-Asp_ww** is reported in Figure 1A (blue curve). The pattern displays few broad reflections indicative of the crystals with nanometric size. The pattern cannot be directly indexed *ab initio*. However, it was compared to that of Al-FUM synthesized as reference as comparative material (Figure 1A, black curve).^38^ The similarities suggested that the two compounds could be isostructural. For this reason, a Pawley refinement of the **Al-l-Asp_ww** pattern was carried out starting from the unit cell of Al-FUM. The refined cell parameters after the Pawley optimization were *a* = 6.8986 Å, *b* = 11.9854 Å, *c* = 14.1402 Å, and β = 123.46° with a cell volume of 975.4 Å^3^ and a chiral *P*2_1_ space group (*R*_wp_: 6%) (Figure S5). These values are very similar to those of Al-FUM and the difference in the unit cell volume was less than 2%. The structure of **Al-l-Asp** was optimized through DFT calculations starting from the Al-FUM framework by properly modifying the organic part from fumaric to l-aspartic acid. Two possible configurations of aspartic acid were tried. One with the amino groups placed on the same side of the chain and the other one with the amino groups on the opposite sides (Figure S6). The two configurations are energetically close to each other, and the calculated diffraction patterns are almost the same. Very likely, aspartate linkers assume a disordered configuration, with the amino groups randomly orientated. The comparison of the experimental and theoretical diffraction patterns is reported in Figure 1B. Although the calculated pattern from the DFT optimized model suggests a good agreement and the reliability of the model found, some differences can be found. For instance, the experimental pattern presents broad and overlapped peaks with respect to the simulated one. The same is also observed when comparing theoretical and experimental patterns of Al-FUM (Figure S7). Furthermore, the reflection observed around 32.5° of 2θ has a lower intensity in the calculated pattern respect to the observed one. This could be due to the fact that the experimental pattern refers to the hydrated **Al-l-Asp_ww** while the calculated pattern refers to the dehydrated phase. A simulation of the pattern of Al-FUM downloaded from Cambridge Structural Database (CSD) was used to get insight into this difference. Figure S7 reports the calculated patterns of the hydrated Al-FUM and the desolvated one. The reduced intensity of the peak around 30° of 2θ for the dehydrated sample can be observed, suggesting that the peak is related to diffuse electron density inside the pores, which could account for the mismatch between the calculated and experimental patterns of Figure 1B. Amino groups are pointing toward the 1D channels, and, from the model, they are not apparently involved in H-bonds with the framework hydroxyl groups [*d*(N···HO) = 3.65 Å]. The square channel size, measured along the atomic centers, measures around 12 × 12 Å^2^, a value similar to that found for Al-FUM (Figure S8).

**Figure 1:**
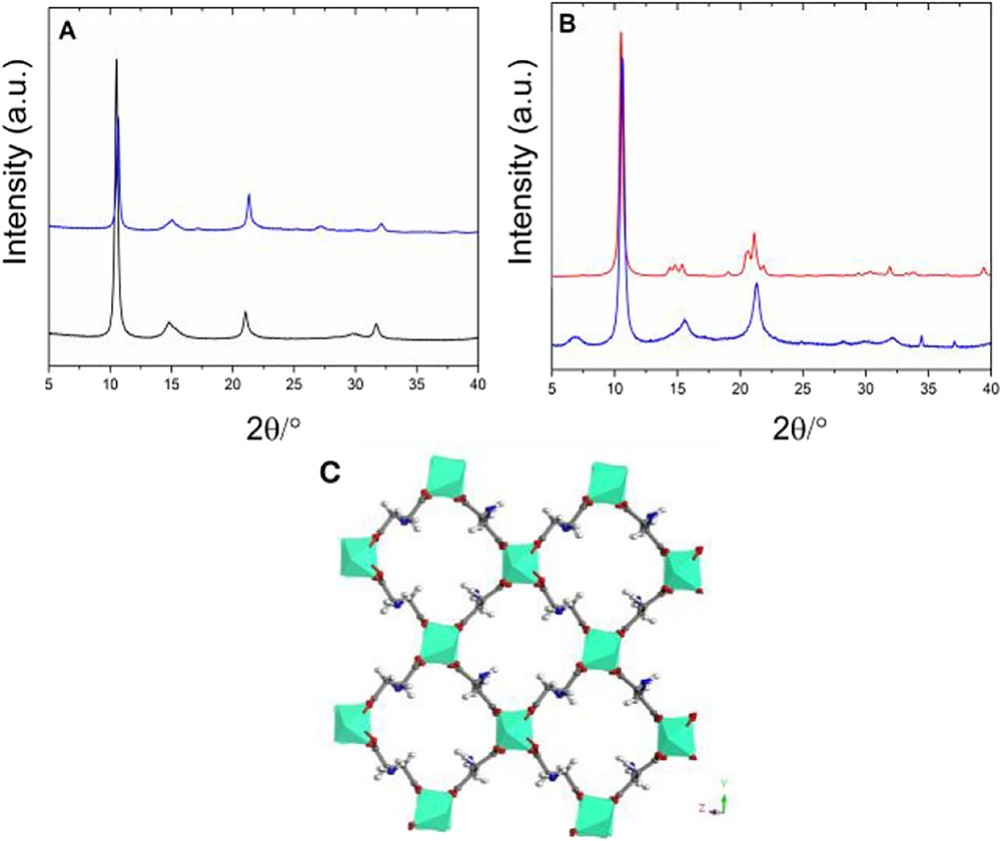
Comparison between experimental PXRD patterns of **Al-l-Asp_ww** (blue) and Al-FUM (black) (A); DFT calculated pattern of **Al-l-Asp** (red) compared with the experimental one (blue) (B); and DFT-simulated crystal structure of **Al-l-Asp** (C). The broad peak around 7.5° of 2θ is due to the hot chamber sample holder.

### ss-NMR Characterization

**Al-l-Asp_ww** was also characterized by multinuclear (^1^H, ^13^C, ^27^Al, and ^15^N) ss-NMR spectroscopy. The {^1^H}-X cross-polarization experiment was used to observe heteronuclei (X = ^13^C, ^15^N), while ^1^H was observed through 2D FSLG HETCOR in the indirect dimension. The spectra were acquired on a sample of **Al-l-Asp_ww** obtained starting with ^15^N-labeled aspartic acid to increase the sensitivity in ^15^N spectra. Figure 2 shows the comparison between the 1D {^1^H}–^13^C cross-polarization spectra (1D {^1^H} ^13^C CP Magic Angle Spinning-MAS) of **Al-l-Asp_ww** at short (50 μs, black curve) and long (1000 μs, red curve) contact times. While playing with the duration of the contact times (short: ca. 50–200 μs; long: ca. 1000 μs and above), it is possible to identify heteronuclei in proximity to protons (e.g., directly bound to protons, short contact) and heteronuclei a few angstroms away (e.g., carbonyls, quaternary carbon, long contact).

**Figure 2:**
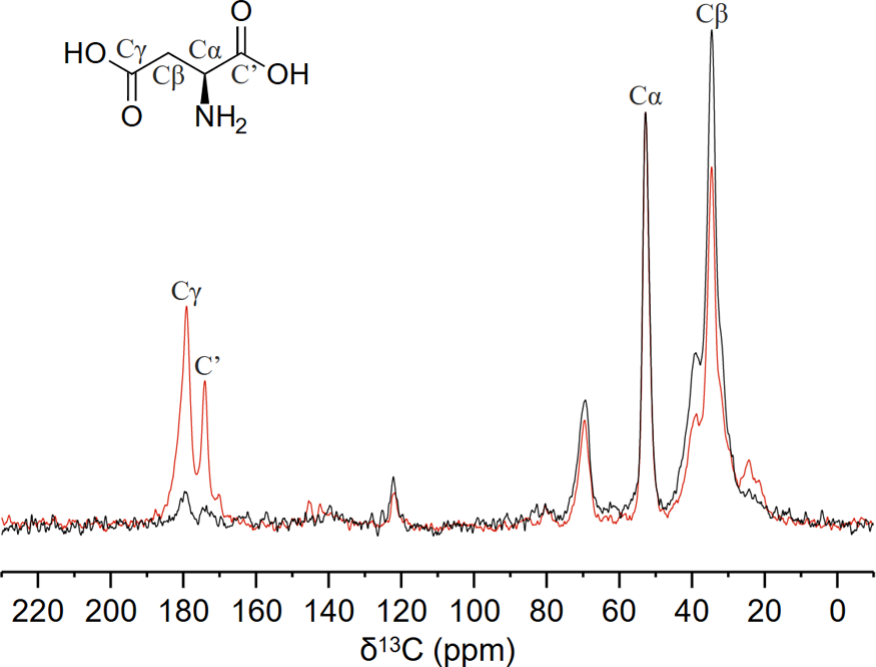
Comparison of 1D {^1^H}–^13^C CP MAS spectra of **Al-l-Asp_ww**, acquired with two different contact times: 1000 μs was chosen as “long contact time” (red), and 50 μs as “short contact time” (black). The spectra were acquired at 20.0 T (850 MHz of proton Larmor frequency) and −3 °C of probe incoming air temperature using 3.2 mm rotor spinning at 20.0 kHz of MAS frequency. Asterisks denote spinning sidebands. The spectra are normalized by setting the same Cα peak intensity.

The acquisition of 2D ^1^H–X FSLG HETCOR spectra (X = ^13^C, ^15^N), by adding the proton dimension is beneficial to distinguish overlapping peaks and get more resolved ^1^H chemical shifts. Figure S9 reports the 2D ^1^H ^13^C FSLG HETCOR spectra at short contact times to assign carbons directly bound to protons and long contact times to assign carbonyls and other quaternary resonances. The ^13^C assignment of the amino acid component of the MOF (Table S1) was determined on the basis of this experimental evidence in CP and HETCOR spectra and the comparison of the experimental chemical shifts with those reported in the Biological Magnetic Resonance Data Bank.^43^ From the shortest contact time 2D ^1^H ^13^C FSLG HETCOR spectrum (i.e., 50 μs, Figure S9B), the Hα (δ_H_ = 3.9 ppm) and Hβ (δ_H_ = 2.5 ppm) peaks could be assigned; they agree with the δ_H_ values reported in the BMRB data bank.

In the 2D ^1^H ^13^C FSLG HETCOR at long contact time (i.e., 1000 μs, Figure S9A), we could observe and assign the quaternary carbons like Cγ and C′. Indeed, Cγ can be distinguished because of the intense cross-peak with the closest proton Hβ, and a much less intense one with further protons like Hα. Conversely, C′ shows a more intense cross peak with the closest Hα and a less intense one for Hβ. This is additional evidence that confirms the δ_C_ values reported in the BMRB database for the assignment of the carbonyls of the amino acid component.

To further investigate the structure of the MOF, a direct excitation (DE) ss-NMR spectrum of ^27^Al, a quadrupolar nucleus (*I* = 5/2) was acquired. Its quadrupolar nature leads to line broadening and chemical shift variations due to the interaction with electric field gradients (EFGs) at the nucleus, but it also provides valuable structural information. The intensity of this interaction is quantified by the quadrupolar coupling constant (*C*_Q_). Figure S10 reports the ^27^Al spectrum showing a strong peak at δ_Al_ ≈ −3 ppm, a shoulder at δ_Al_ ≈ −18 ppm, and another peak at δ_Al_ ≈ 5 ppm. To obtain more detailed information about the ^27^Al sites, we performed a quadrupolar fitting with MrSimulator^44^ to extract the isotropic chemical shift (^iso^δ), the nuclear electric quadrupolar coupling constant (*C*_Q_) and the asymmetry parameter (η_Q_) that describes the degree of asymmetry of the EFG.

The fit was obtained with two main components (Figure 3) suggesting the presence of a distribution of coordination sites on the MOF, with at least three possible species present. The ^iso^δ values, of about 17 ppm are compatible with six-coordinate ^27^Al sites.^45,46^ The two components shows different the *C*_Q_ values of 9.09 and 12.0 MHz (Table S2), and are considerably broadened, the latter is an indication of the heterogeneity in the sample that agrees with the distribution of orientation of the aspartate ligand coordinating either with the C′ site or the Cγ site. The two different species can be attributed to different level of hydration in the MOF pores, as already observed in ^27^Al spectra in similar MOFs.^38,47^ As observed in the literature, the more hydrated form has a larger *C*_Q_ and a larger line broadening. This additional line broadening is consistent with a larger heterogeneity that accumulate contribution coming from the hydration to that indicated above from the linker orientation.

**Figure 3:**
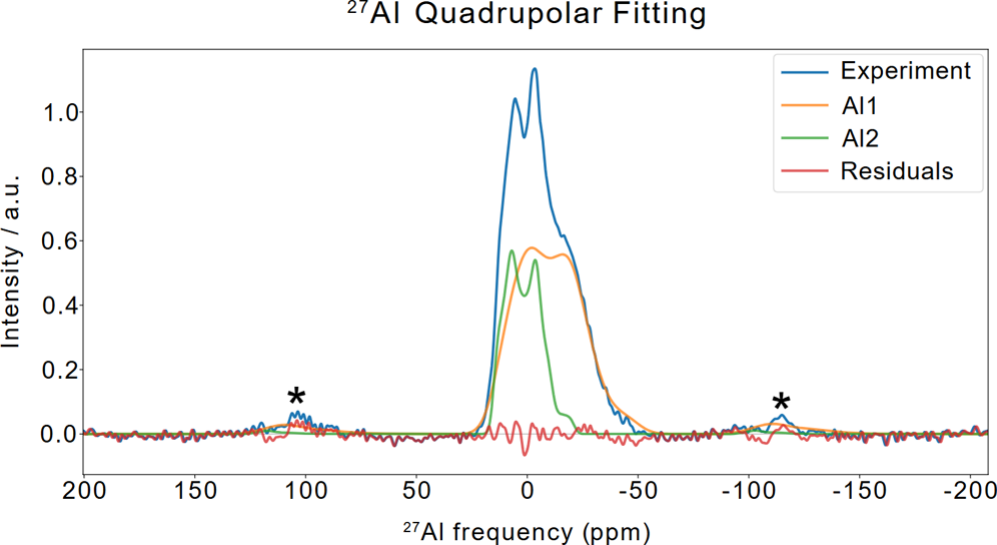
Quadrupolar fitting of 1D ^27^Al Hahn-Echo MAS spectrum of **Al-l-Asp_ww** done with the software MrSimulator.^44^ The spectrum was acquired at 16.4 T (700 MHz of proton Larmor frequency) using 3.2 mm rotor spinning at 20 kHz of MAS frequency as reported in Figure S9. The final fit was achieved through two iterations cycles.

To better confirm this analysis, we have also acquired a 2D ^27^Al MQMAS spectrum (3QMAS) on the same sample as reported in Figure S11. This 2D spectrum shows in the isotropic dimension F1 two resonances corresponding to the different species fitted in Figure 3, with one resonance more broadened than the other by a larger *C*_Q_ values, confirming the analysis done above on the 1D spectrum. These are consistent with the Al coordination expected for this MOF and indicate a good quality of the preparation expanding on the knowledge derived from X-ray diffraction data.

### Optimization of the Activation Procedure and Textural Properties

The textural properties were evaluated through N_2_ sorption analysis once the optimal activation conditions were established by in situ IR spectroscopy analyses. The as-synthesized **Al-l-Asp_ww** IR spectrum (Figure 4A, light blue curve) showed a broad band around 3700–3000 cm^–1^, indicating occluded pores. Upon outgassing RT with dynamic ultrahigh vacuum, these bands decreased in intensity but were still prominent, suggesting that heating was necessary to properly clean the channels of this MOF. First, the sample was activated at 150 °C overnight (labeled as **Al-l-Asp_150**, where 150 refers to the temperature of the treatment), but as shown in the dark blue line spectrum, bands associated with solvent residue were still present. It is worth noting that (as in Al-FUM) **Al-l-Asp** is made of μ_2_(OH) species bridged to two adjacent Al­(III) ions. From the DFT-optimized structure, these OH species are not involved in H-bonding interactions with the amino group, meaning that a sharp isolated signal centered around 3700 cm^–1^ (related to the stretching mode of structural OH species bridging two Al atoms)^38,47^ should be expected. The absence of such a signal, only notable as a weak shoulder at 3700 cm^–1^, suggested that the structural OH groups were still interacting with undefined species still trapped within the MOF channels. The activation temperature was increased up to 200 °C, after the second weight loss from TGA, but before the starting point of the MOF decomposition, as further confirmed by VT-PXRD (Figures S2 and S3). The final spectrum of **Al-l-Asp_200** still showed broad signals covering the range from 3500 to 2500 cm^–1^ (related to the ν­(NH) and ν­(CH) stretching modes), but the ν­(OH) vibrational mode was sharp in this case as expected. As reported by Abodunrin et al. for the synthesis of zirconium aspartate MIP-202, the use of l-aspartate led to a cationic metal organic framework where the amino group on aspartate was protonated and NO_3_^–^ acted as an extra-framework counterion.^48^ In order to check if this also occurred with aluminum, the presence of NH_3_^+^ species and NO_3_^–^ counterions was simulated through DFT. The simulated vibrational frequencies of the ideal MOF (**Al-l-Asp_ww**) and the cationic one (**Al-l-Asp-NH_3_^+^**) were compared to the experimental IR spectrum. As clearly shown in Figure 4B, the experimental IR spectrum of **Al-l-Asp** (green curve) had a better agreement with the calculated frequencies than that of cationic framework **Al-l-Asp-NH_3_^+^**. In fact, the ideal **Al-l-Asp** form should exhibit only a few defined O–H stretching modes in the high-frequency bound of the spectrum (associated with unperturbed and weakly perturbed μ_2_–OH of the inorganic chain), whereas a broad envelope of signals is observed experimentally. The latter is better qualitatively reproduced by the simulated spectrum of **Al-l-Asp-NH_3_^+^**, which features multiple intense signals in this spectral region, due to stretching modes of both μ_2_–OH and NH_3_^+^ moieties involved in a complex network of H-bonds. It is worth noticing that the aforementioned calculation led to enforcing the conclusion of the presence of a cationic framework with a counterion blocked in the channels (fundamental to stabilize the charge of the system); however, this analysis cannot confirm the presence of NO_3_^–^ since no related IR signals are visible in the spectra.

**Figure 4:**
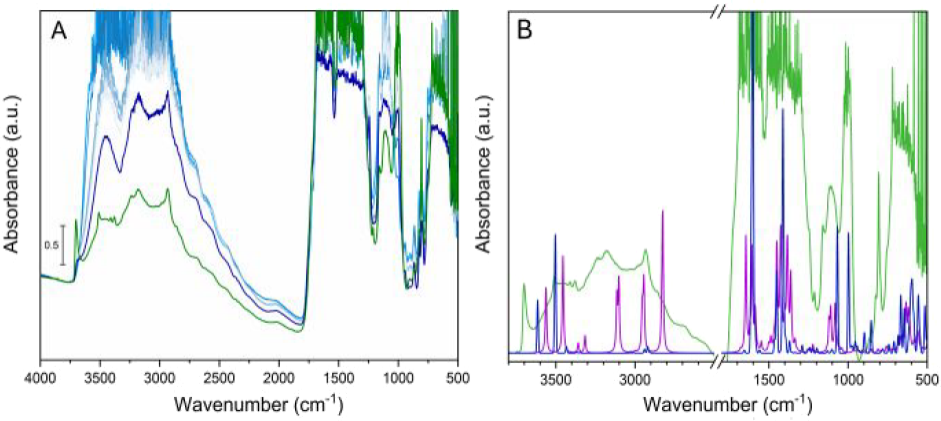
IR spectra of **Al-l-Asp_ww**: as synthesized (blue), outgassing sequence at RT (light blue curves), activated at 150 °C (dark blue) and activated at 200 °C (green) (A), comparison between the experimental spectrum of activated **Al-l-Asp_200** (green curve) and simulated IR spectra. The magenta spectrum simulates the IR spectrum of **Al-l-Asp-NH_3_^+^**, while the blue spectrum represents the simulated spectrum of the ideal **Al-l-Asp** structure (B).

To experimentally investigate the protonation state of the amine group in the aspartate MOF, we used solid-state ^15^N NMR spectroscopy; the sample was prepared using ^15^N-labeled aspartate because of the very low isotopic natural abundance of ^15^N (0.3%). In the 1D {^1^H}–^15^N CP MAS spectrum (Figure 5), we observe a main resonance at δ_N_ = 36 ppm compatible with the shift of the protonated amine group of l-Asp (R–NH_3_^+^),^49^ evidencing the MOF acidic environment. The resonance is comparably broad and should reflect the distribution of orientations of the amino group in the MOF structure. Another small peak is observed in the direct excitation (DE) spectrum around δ_N_ = 39 ppm, indicating a small amount of a second species, highly mobile, nonvisible in the CP spectrum. This sharp peak could be due to a small amount of free aspartate molecules remaining trapped inside the MOF structure and replacing, with their negative charge, the anionic counterion.

**Figure 5:**
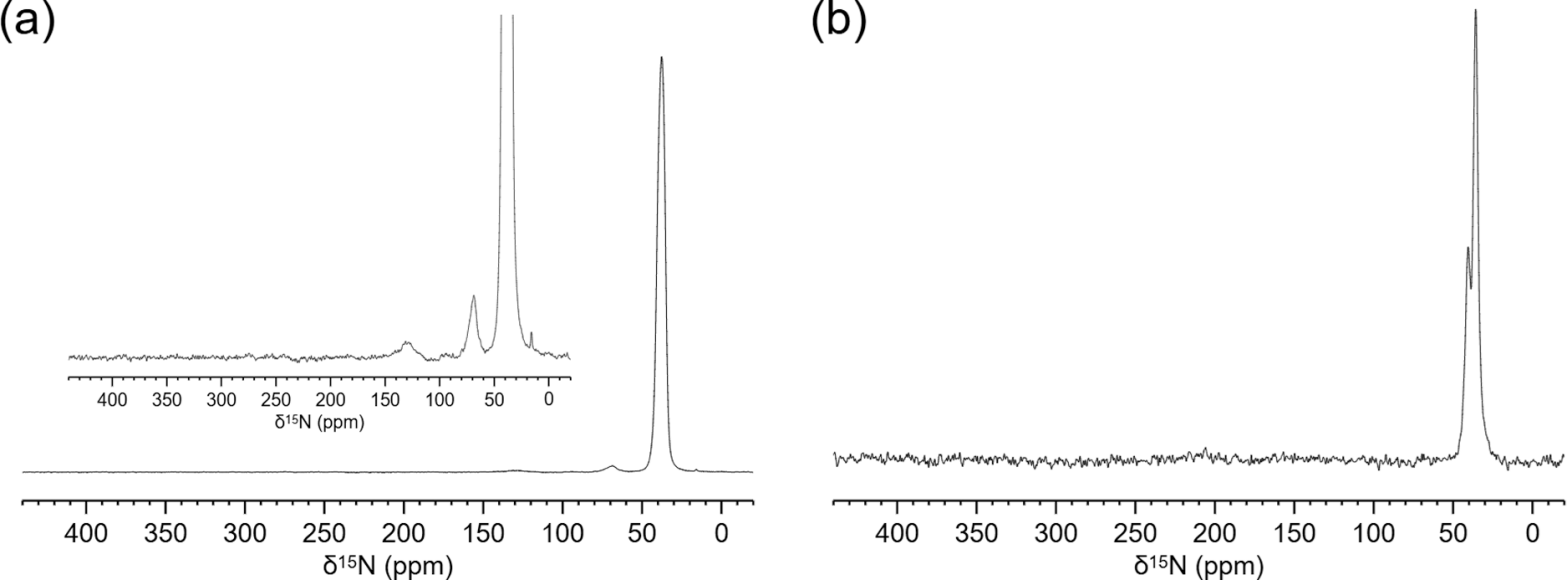
1D {^1^H} ^15^N CP MAS long contact time (1500 μs) (a), and 1D ^15^N MAS direct excitation (b) spectra (DE) of **Al-l-Asp_ww** acquired at 20.0 T (850 MHz of proton Larmor frequency), using 3.2 mm rotor spinning at 20 kHz of MAS frequency and −3 °C of probe incoming air temperature. To detect possible traces of nitrate, the spectra were acquired for 3 d 6 h (a) and 3 d 19 h (b).

Despite the long acquisition times (detailed in Figure 5 caption), the presence of nitrate ion (expected at δ_N_ ≈ 380 ppm)^50^ was not observed in either CP or direct excitation spectra. This could not completely exclude the presence of nitrate but suggests that the workup procedure removed a large part of this species or that other species act as extra framework counterions. To further investigate the presence of nitrate ions, elemental analysis was performed on **Al-l-Asp_ww**. The ideal ratio for aspartic acid is C/N = 4 whereas in our case we have an excess of N that we can evaluate through the formula C/N = 4/(1 + *x*) = 1.79/0.68 = 2.91. Solving *x*, we get *x* = 0.31. This excess of N can be attributed to the nitrate counterion. The new formula is now [Al­(OH)­(Aspartate)­(HNO_3_)_0.31_]·1.5H_2_O (FW = 225.3 g·mol^–1^). The calculated wt % are now in good agreement with the experimental values (all wt % values are reported in Table S3). TGA analysis also confirms the calculated weight from the analysis, taking as reference 0.5 Al_2_O_3_ at the end of the analysis (22% residue) leading to a calculated FW of 208 g·mol^–1^, calculated from the formula [Al­(OH)­(Aspartate)­(HNO_3_)_0.31_] (FW = 198 g·mol^–1^).

In the CP spectrum in Figure 5A, it can be observed small weak signals at δ_N_ ≈ 70 ppm that are consistent with very small amount of carbamate nitrogen probably due to incorporation of traces of atmospheric CO_2_.^51^

The 2D ^1^H–^15^N FSLG HETCOR spectra (Figure S11) confirmed that the ^15^N and ^1^H chemical shifts (δ_N_ ≈ 36 ppm, δ_H_ ≈ 8 ppm) of the aminic moiety are consistent with a protonated species, which is expected given the pH conditions used during the synthesis.

The textural properties of **Al-l-Asp** MOF were analyzed by collecting adsorption isotherms using N_2_ as adsorptive at its liquefaction temperature (−196 °C) (Figure 6). Prior to the analysis, the sample was first activated overnight at 150 °C. The resulting curve is characterized by a very low microporous volume, considering that the isotherm reaches saturation with 5 cm^3^·g^–1^ (blue curve). The resulting BET surface area of **Al-l-Asp_150** is 19.19 ± 0.04 m^2^·g^–1^. This confirmed that the material was not completely activated at 150 °C and that the pores were still occluded. A second isotherm was collected by activating the material to 200 °C (green curve). This time, the resulting isotherm showed a much larger N_2_ uptake at saturation and the typical profile of a type I isotherm.^52^ The resulting BET area of **Al-l-Asp_200** is 598 ± 1 m^2^·g^–1^.

**Figure 6:**
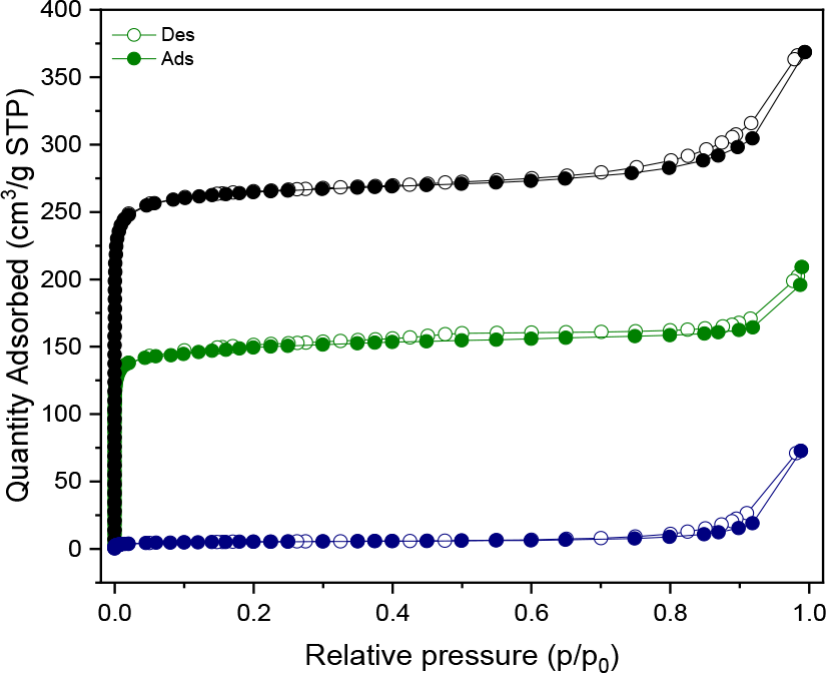
N_2_ adsorption (full symbols) and desorption (empty symbols) isotherm collected at −196 °C on: **Al-l-Asp_150** (blue curve), **Al-l-Asp_200** (green curve), and Al-FUM (black curve).

The pore analysis was conducted only on the sample activated at 200 °C (**Al-l-Asp_200**). Nonlocal DFT (NL-DFT) was employed by choosing the fit with the lowest standard deviation value. As depicted in Figure S12A (green curve), the PSD showed a unique family of pores centered around 0.6 nm, indicating a uniform ultramicroporous structure. The CPV (Figure S12B, green curve) reached a plateau around 0.33 cm^3^·g^–1^. As a reference material, Al-FUM was also investigated by collecting its N_2_ adsorption isotherm at −196 °C. Prior to analysis, the sample was activated overnight at 100 °C, as reported in the reference patent (black curve).^6^ As expected, the area of Al-FUM is higher than that of **Al-l-Asp_200**, resulting in a value of 1066 ± 2 m^2^·g^–1^, in good agreement with the value already reported in the literature.^38^ NL-DFT was also performed. The resulting PSD (Figure S12A, black curve) shows a single family of pores centered at 1.06 nm, which are larger than those of **Al-l-Asp_200**, as expected, given the absence of the amino group in the former. Despite the presence of amino groups pointing to the channels of the pores, the **Al-l-Asp_200** SSA is much lower than expected in comparison to Al-FUM, considering their almost negligible unit cell volume differences. It can be concluded that the lower SSA could be indicative of counterions partially obstructing the SSA of the MOF. To conduct a more precise analysis on the real reduction of the accessible surface area of **Al-l-Asp** solely induced by the presence of amino groups pointing within the pores, pore analysis was conducted by using the software Zeo++ on Al-FUM and the simulated structure of **Al-l-Asp**. The calculated surface areas were found to be 1341 and 942 m^2^·g^–1^ (probe radius of 1.8 Å). The slightly higher surface area of Al-FUM in comparison with the experimental value may be attributable to the fact that Zeo++ is solely a geometric-based analysis. In this analysis, the probe is regarded as purely spherical, and no interaction with the surface of the adsorbent is taken into consideration. Theoretical calculations indicate that the **Al-l-Asp** has an SSA that is 30% lower than that of Al-FUM. However, the discrepancy between the calculated isotherms is found to be 44%.

Since the analysis of the textural properties revealed that the activation at 200 °C was pivotal to free the channels of **Al-l-Asp**, further techniques were employed to investigate the species eliminated during this thermal treatment. For this reason, a second TG analysis was performed by coupling a mass spectrometer (MS). From the MS, the signal attributed to ammonia (*m*/*z* = 17 amu) and to a fragmentation peak of fumaric acid (*m*/*z* = 45 amu) were observed at *T* = 200 °C, which corresponds to a significant weight loss of the TG curve (Figures S2 and S13). These two signals are ascribed to the decomposition of aspartic acid at that temperature, releasing ammonia and fumaric acid.

ss-NMR analysis was also carried out on the sample treated at 200 °C (**Al-l-Asp_200**) to get better information on the framework stability. The 1D {^1^H}–^13^C CP MAS (Figure 2) and 2D ^1^H ^13^C FSLG HETCOR (Figure S9) spectra reveal that traces of other species are present in the pore cavities of the activated MOF. The weak resonances around δ_C_ ≈ 26.3 and ≈22 ppm correlating with proton species at δ_C_ ≈ 2 ppm are compatible with aliphatic signals from residual GVL, which is also confirmed by the ^13^C direct excitation spectra in Figure S14. The very weak signals centered at δ_C_ = 138 ppm suggest the presence of a small amount of sp^2^ carbon atoms with directly bound alkenyl protons and are consistent with chemical shifts from dehydrogenated derivatives of l-aspartic acid: fumaric and/or maleic acid.^53^ The resonance centered at [δ_C_ = 70 ppm; δ_H_ = 3.9 ppm], as well as the resonance at [δ_C_ = 40 ppm; δ_H_ = 2.1 ppm] are compatible with the C–OH and with the CH_2_ of malic acid,^54^ respectively, probably generated upon hydration of the dehydrogenated l-Asp derivatives. The presence of GVL, malic, fumaric, or maleic acid is also suggested by the presence of small peaks in the carbonyl region around 170 ≤ δ_C_ ≤ 180 ppm in the long contact time spectra. These resonances are minor resonances compared to those of the MOF framework. Anyway, a detailed analysis of these impurities is reported in the Supporting Information through the solution NMR spectra of the MOF digested with a small amount of hydrofluoric acid (HF).

### CO_2_ Adsorption Properties

CO_2_ isotherms of **Al-l-Asp_200** were collected at three different temperatures: 0, 10, and 20 °C (Figure S157A) for the evaluation of the isosteric heat of adsorption (*Q*_st_) by applying the Clausius–Clapeyron equation (see eq 1). The CO_2_ adsorption isotherms exhibited a typical type I behavior, characteristic of microporous materials.^52^ **Al-l-Asp_200** showed a relatively high CO_2_ uptake at *p*_CO_2__ = 1 bar, reaching approximately 3 mmol·g^–1^ at *T* = 0 °C, 2.5 mmol·g^–1^ at *T* = 10 °C and about 2 mmol·g^–1^ at *T* = 20 °C. Negligible hysteresis revealed a reversible CO_2_ physisorption mechanism. To better compare the CO_2_ adsorption performances with those of Al-FUM, we also measured the CO_2_ isotherms for the latter. The comparison between the two CO_2_ isotherms at *T* = 0 °C is reported in Figure 8A. While Al-FUM adsorbs up to 4 mmol·g^–1^ (a value higher than that of **Al-l-Asp_200**), this latter has anisotherm shape with a steep increase in the low-pressure region suggesting higher interaction with CO_2_. The uptake measured at *p*_CO_2__ = 0.15 bar (a pressure value considered as typical for postcombustion applications) and *T* = 0 °C was around 1.5 mmol·g^–1^ (Figure 8A). At a higher temperature (20 °C), the value drops to 0.8 mmol·g^–1^. Despite this value being sensibly lower than those reported for postcombustion CO_2_ uptake benchmark MOFs such as CALF-20,^55^ MIL-120,^56^ UTSA-16 ^57^ and ALF,^58^ it has to be underlined that it is higher than that measured for Al-FUM at 20 °C (about 0.5 mmol·g^–1^).^7^ The CO_2_ isosteric heat of adsorption of **Al-l-Asp_200** (Figure 7B) at 0.01 mmol·g^–1^ coverage is approximately 30 kJ·mol^–1^; this value is indicative of a physisorption mechanism, suggesting moderate interaction strength between CO_2_ and the framework.^59^ *Q*_st_ is lower than the average value measured for physisorption amine-free benchmark MOFs for postcombustion (around 40 kJ·mol^–1^),^7^ possibly due to the presence of residual NH_3_^+^ species together with free amine. However, **Al-l-Asp_200** exhibited a significantly higher CO_2_ heat of adsorption than Al-FUM (30 vs 21 kJ·mol^–1^, respectively) indicating a stronger interaction with CO_2_.^7^ Such stronger interaction is still ascribable to a physisorption process (possibly implying O = C=O···H–N H-bonding interactions between CO_2_ and the amino groups), as the formation of chemisorbed species at the amine functionality (i.e., carbamates/carbamic acid) would imply a significantly higher adsorption heat (typically in the 50–100 kJ·mol^–1^ range).^60–63^ As a plus, the relatively low *Q*_st_ value of **Al-l-Asp_200** facilitates an easy regeneration process.^59^ The ease of regeneration, which does not require any additional thermal treatment, prevents any further degradation of the framework. This was demonstrated by repeating the CO_2_ adsorption isotherms at 0 °C three times. Prior to the first cycle, the workup procedure consisted of activating at 200 °C to free the pores of the MOF. On the contrary, before the second and third cycles, the regeneration only consisted of evacuation at room temperature. The three isotherms superimpose, demonstrating that the material is fully regenerated after each cycle and no further degradation occurs.

**Figure 7:**
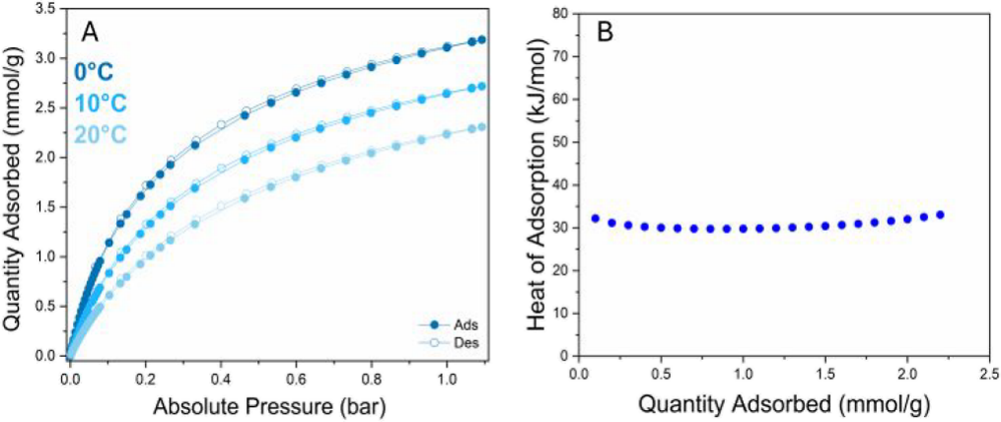
CO_2_ adsorption/desorption isotherms at *T* = 0, 10 and 20 °C (A); CO_2_ heat of adsorption of **Al-l-Asp** (B).

It is evident that the calculated *Q*_st_ value is typical of a physisorption mechanism. However, it is slightly larger than the heat of adsorption generally calculated for MOFs containing aromatic amines such as 2-aminoterephtalate (BDC-NH_2_^2–^) in MIL-53­(Al)-NH_2_ and UiO-66-NH_2_. The partial delocalization of the nitrogen lone pair on the aromatic ligand reduces its basicity.^9,64^ Indeed, it has been documented that the CO_2_ heat of adsorption for UiO-66-NH_2_ is 25 kJ·mol^–1^, comparable to that of pristine UiO-66.^65^

The IAST selectivity of CO_2_ over N_2_ (Figure 8B) was evaluated for **Al-l-Asp_200** at *T* = 0 °C using the IAST theory that predicts multicomponent isotherms starting from the experimental single-component isotherms. The selected mixture composition for the IAST calculation is that typically found in coal-derived flue gas (15% CO_2_–85% N_2_); under these conditions, the CO_2_/N_2_ selectivity remains relatively high across the investigated pressure range (0.1–1 bar), suggesting a strong preferential adsorption of CO_2_ over N_2_ even under diluted conditions. As the pressure increases, selectivity exhibits a gradual upward trend, reaching a value of 86 at 1 bar. The consistently high selectivity observed across the pressure range underscores the potential of the MOF as an efficient material for CO_2_ capture and separation. These results were compared with those of other similar Al-based MOF previously reported in the literature, such as Al-FUM and Al-TFS developed by our group.^47^ Notably, **Al-l-Asp_200** exhibits significantly higher selectivity compared to both Al-FUM and Al-TFS. These findings underscore the promising potential of **Al-l-Asp_200** for application in CO_2_/N_2_ separation processes, particularly in postcombustion capture scenarios.

**Figure 8:**
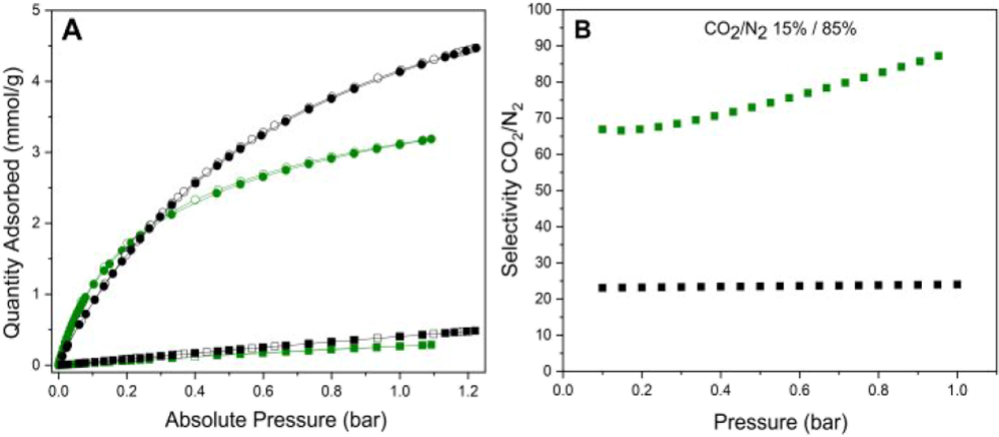
CO_2_ (circle points) and N_2_ (square points) adsorption/desorption isotherms at *T* = 0 °C of: **Al-l-Asp** (green) and Al-FUM (black) (A); ideal adsorbed solution theory (IAST) selectivity for a CO_2_/N_2_ 15:85 mixture was calculated in the pressure range of 0.1–1 bar starting from the simulation of mixed gas isotherms obtained by using the IAST++ software both for **Al-l-Asp** (green) and Al-FUM (black) (B).

## Conclusions

A novel aluminum MOF based on l-aspartic acid, structurally homologue to Al-fumarate basolite A520, has been synthesized and thoroughly characterized through several solid-state techniques. The use of GVL as a more sustainable solvent is crucial for the obtainment of a nanocrystalline material in pure form. Two structural models with different orientations of the amino groups were proposed by means of DFT analysis coupled with PXRD Pawley refinement of the unit cell. FT-IR and ss-NMR spectroscopies indicated partial protonation of the amino groups, charge-compensated by nitrate anions trapped in the channels, which hampers a direct interaction of the −NH_2_ groups with CO_2_. Solid-state and solution NMR analysis on the digested MOF showed the presence of fumaric acid (not present in the pristine material), possibly coming from a partial linker decomposition at the temperatures required to access the porosity of the system. The MOF exhibits a better affinity toward CO_2_ compared to Al-FUM and an IAST CO_2_/N_2_ selectivity comparable to those of benchmark MOFs used for postcombustion gas upgrade. However, it must be emphasized that this approach remains an approximation and that a rigorous assessment of separation performance would require breakthrough experiments to account for kinetic effects. Further development will be devoted to modulating the synthesis and activation conditions of **Al-l-Asp** and the MOF testing in real gas mixture purification experiments.

## Supplementary Material



## Notes

PXRD patterns, SEM images, TGA/MS analysis, VT-PXRD patterns, NMR spectra, CO_2_ isotherms, Pawley refinement of the unit cell, ss-NMR instrumental parameters (PDF)
