# Supplementary material for: Structure and Carbon Dioxide Adsorption Properties of a Nanosized Aluminum l‑Aspartate Metal–Organic Framework
Source: ACS Appl Nano Mater. 2026 Apr 21;9(17):7608–18. doi: 10.1021/acsanm.6c00412 (PMC13142191; doi:10.1021/acsanm.6c00412)
Supplement: Supplementary file 1 [file an6c00412_si_001.pdf]

Supporting information

Structure and Carbon Dioxide Adsorption Properties of a Nanosized Aluminum L-Aspartate Metal-Organic Framework

Letizia Trovarelli[a,b,‡] *Virginia Guiotto*[b*,‡] *Maria Sole Notari*[a], *Lorenzo Isidoro*[a,c], *Giacomo Provinciali*[c], *Concetta Bafaro*[a], *Andrea Rossin*[c*], *Martino Degli Innocenti*[d,e], *Naomi Anna Consoli*[d,e], *Moreno Lelli*[d,e*] *Marco Taddei*[f],*Matteo Signorile*[b], *Valentina Crocellà*[b] *and Ferdinando Costantino*[a*]

a Dipartimento di Chimica, Biologia e Biotecnologie (DCBB). Università di Perugia, Via Elce di Sotto 8, 06123 Perugia (Italy)

b Dipartimento di Chimica, Centro di Riferimento NIS, Unità di Ricerca INSTM, Università degli Studi di Torino, Via G. Quarello 15/A and Via P. Giuria 7,I-10125 Torino, Italy

c Istituto di Chimica dei Composti Organometallici (CNR-ICCOM), Via Madonna del Piano 10, Sesto Fiorentino (Firenze), 50019, Italy.

d Centre of Magnetic Resonance (CERM) Università di Firenze, Via Luigi Sacconi 6, Sesto Fiorentino (Firenze), 50019, Italy.

e Dipartimento di Chimica Ugo Schiff, Università di Firenze, Via della Lastruccia 3-13, Sesto Fiorentino (Firenze), 50019, Italy.

f Dipartimento di Chimica e Chimica Industriale, Unità di Ricerca INSTM, Università di Pisa, Via Giuseppe Moruzzi 13, 56124 Pisa, Italy

Table of Contents

1. Synthesis and preliminary characterization 2

2. DFT Calculations and structural description 4

3.Solid-state NMR (ssNMR) Characterization. 5

4. Optimization of the activation procedure and textural properties 8

5. CO_2 adsorption properties 10

6.Tables of NMR Experimental Parameters 11

7.Solution NMR Analysis of the digested **Al-L-Asp** 18

1. Synthesis and preliminary characterization

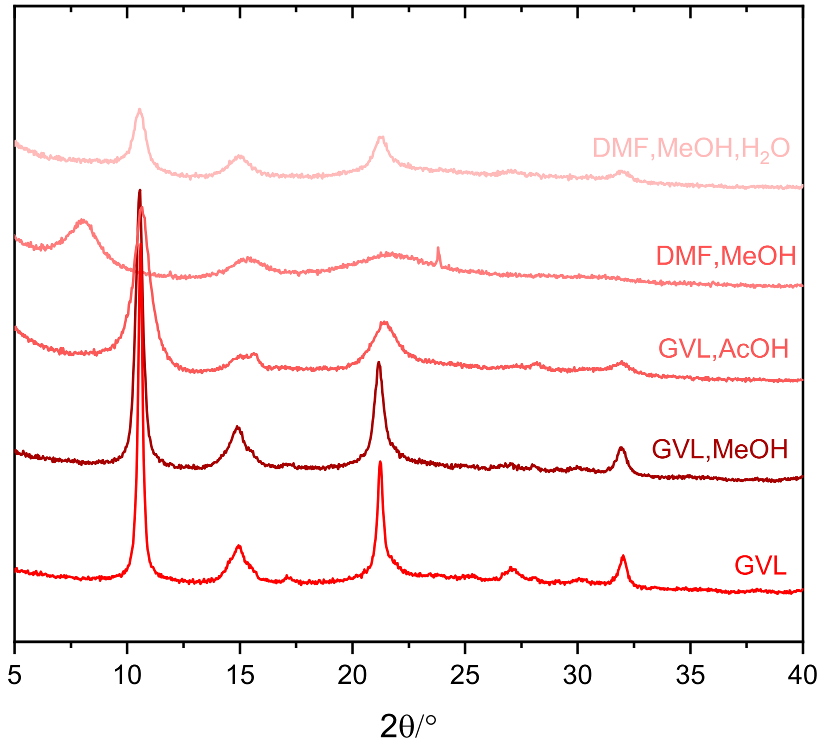

Figure S1: PXRD patterns of **Al-L-Asp** synthesized with different solvents, showing that only the MOF obtained in GVL yields the desired material.

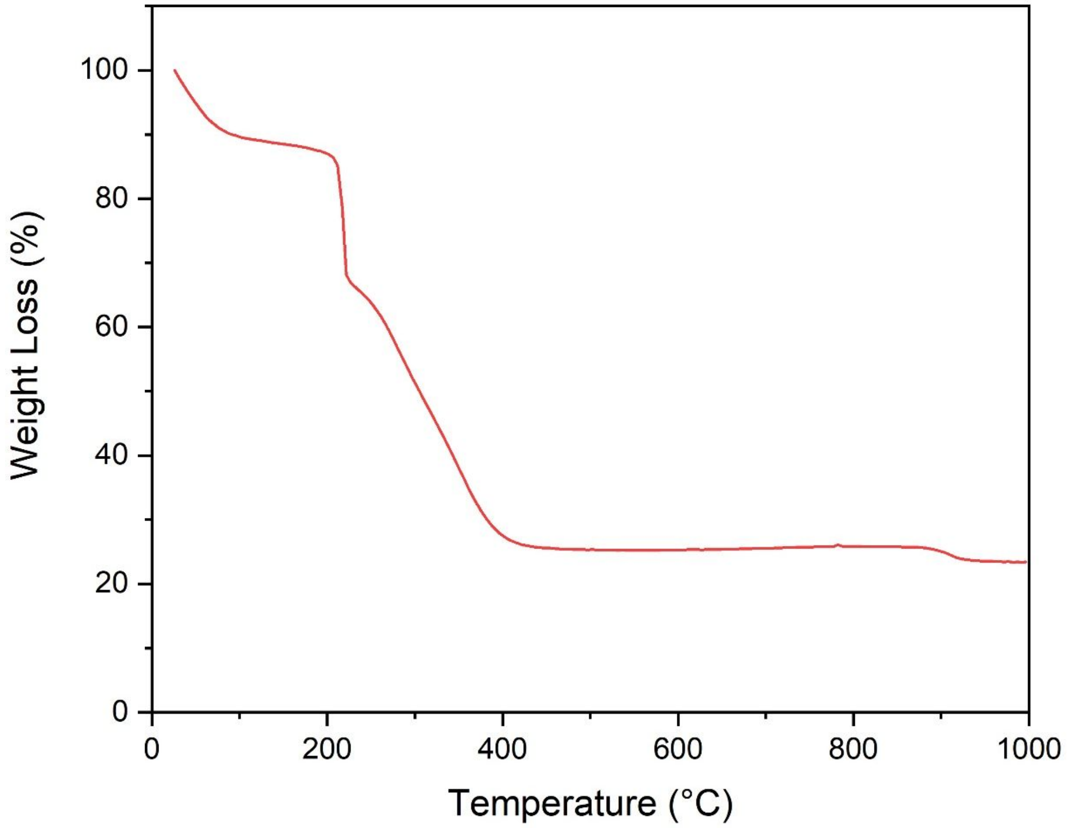

Figure S2: TG curve of **Al-L-Asp_ww**.

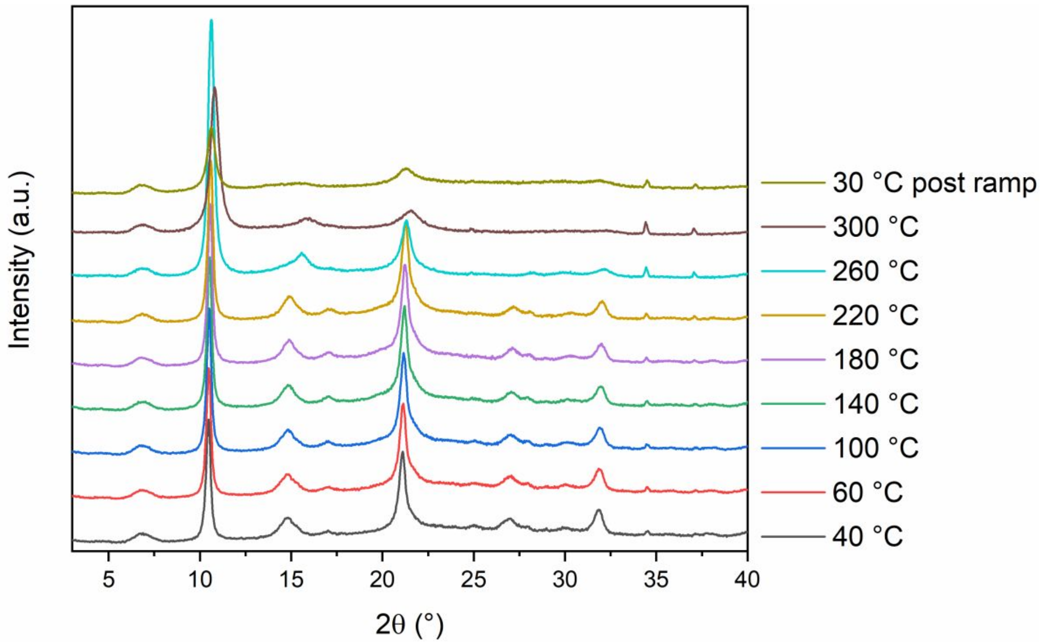

Figure S3. VT-XRPD patterns for **Al-L-Asp_ww** (the broad peak around 7 ° of 2θ belongs to the hot chamber sample holder)

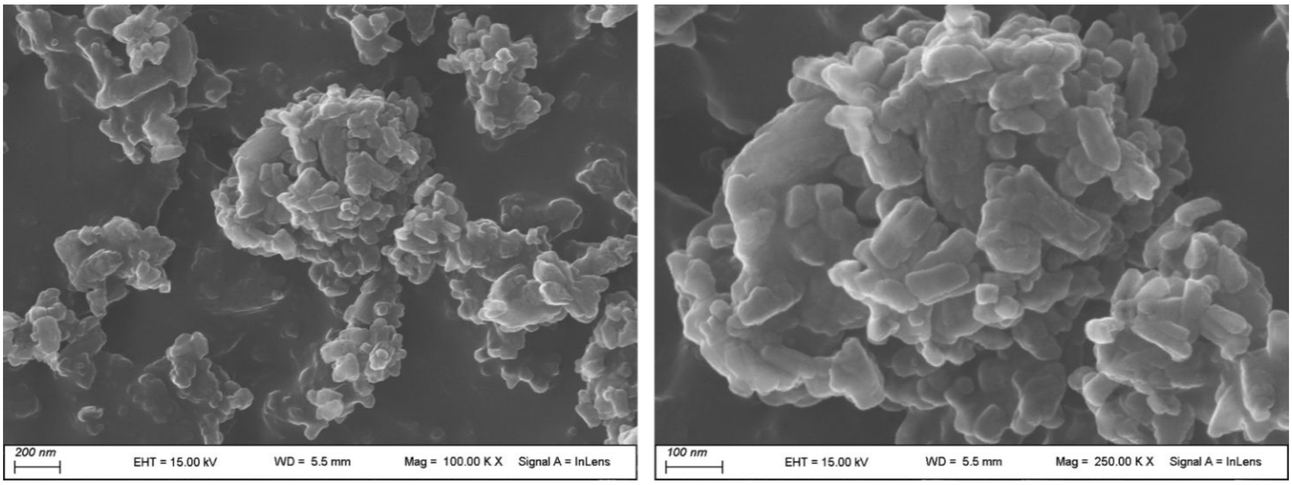

Figure S4. SEM images of **Al-L-Asp_ww** at different magnification

2. DFT Calculations and structural description

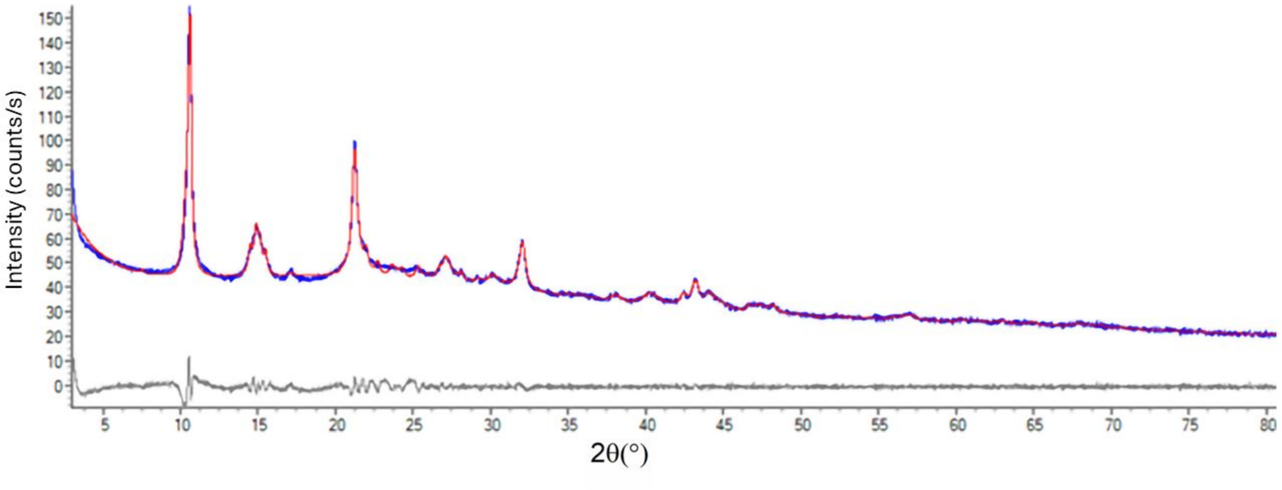

Figure S5. Pawley refinement of **Al-L-Asp_ww**. Blue: experimental pattern; red: calculated pattern; grey: difference.

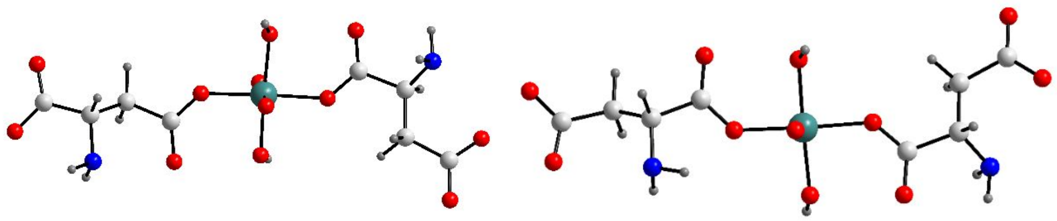

Figure S6. Asymmetric unit of **Al-L-Asp** with the amino group placed on the same side (left) and on the opposite side (right) of the chain. Colour code: C (white); H (grey); N (blue); O (red); Al (petrol green).

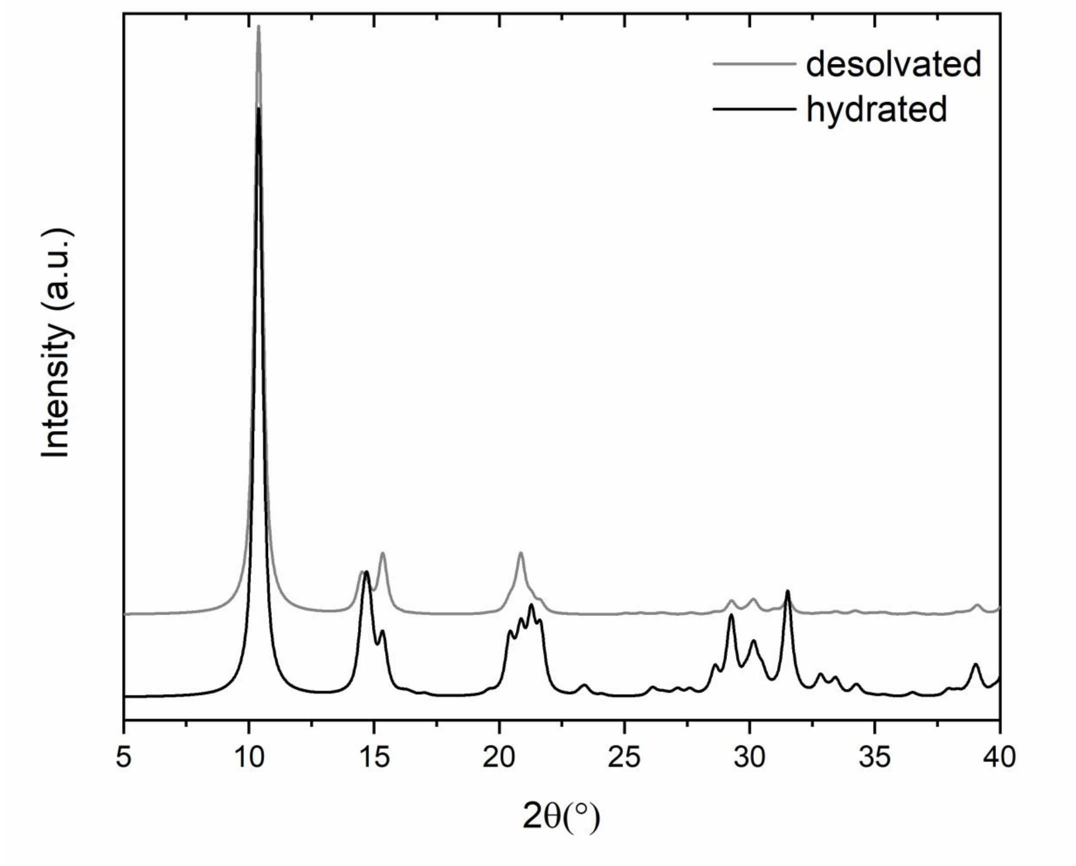

Figure S7. Comparison between the simulated PXRD patterns of Al-FUM in its hydrated (black curve) and desolvated (grey curve) phases

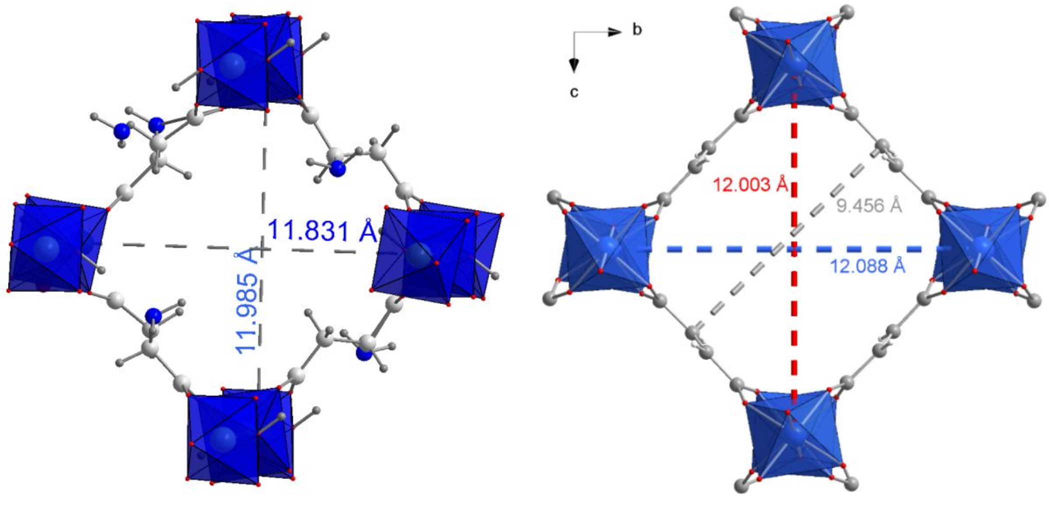

Figure S8. Comparison between the channel size of **Al-L-Asp** (left) and Al-FUM (right)

3. Solid-state NMR (ssNMR) Characterization.

Table S1. ^{13}C NMR assignment of the MOF Al-L-Asp_ww resonances.

**Cγ**	**δ_C = 179 ppm**
**C'**	**δ_C = 174 ppm**
**Cα**	**δ_C = 52 ppm**
**Cβ**	**δ_C = 35 ppm**

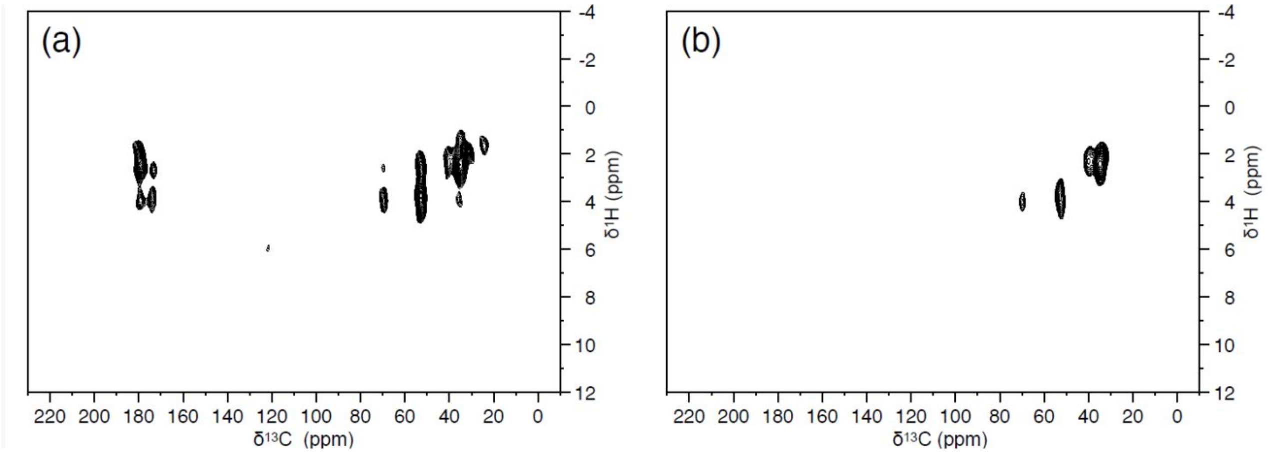

Figure S9. 2D ^1H-^{13}C-FSLG HETCOR spectra of **Al-L-Asp_ww** acquired with two different contact times: 1000 μs and 50 μs, respectively (a) and (b). The spectra were acquired at 20.0 T (850 MHz of proton Larmor frequency) and 270 K of probe incoming air temperature, using 3.2 mm rotor spinning at 20.0 kHz of MAS frequency.

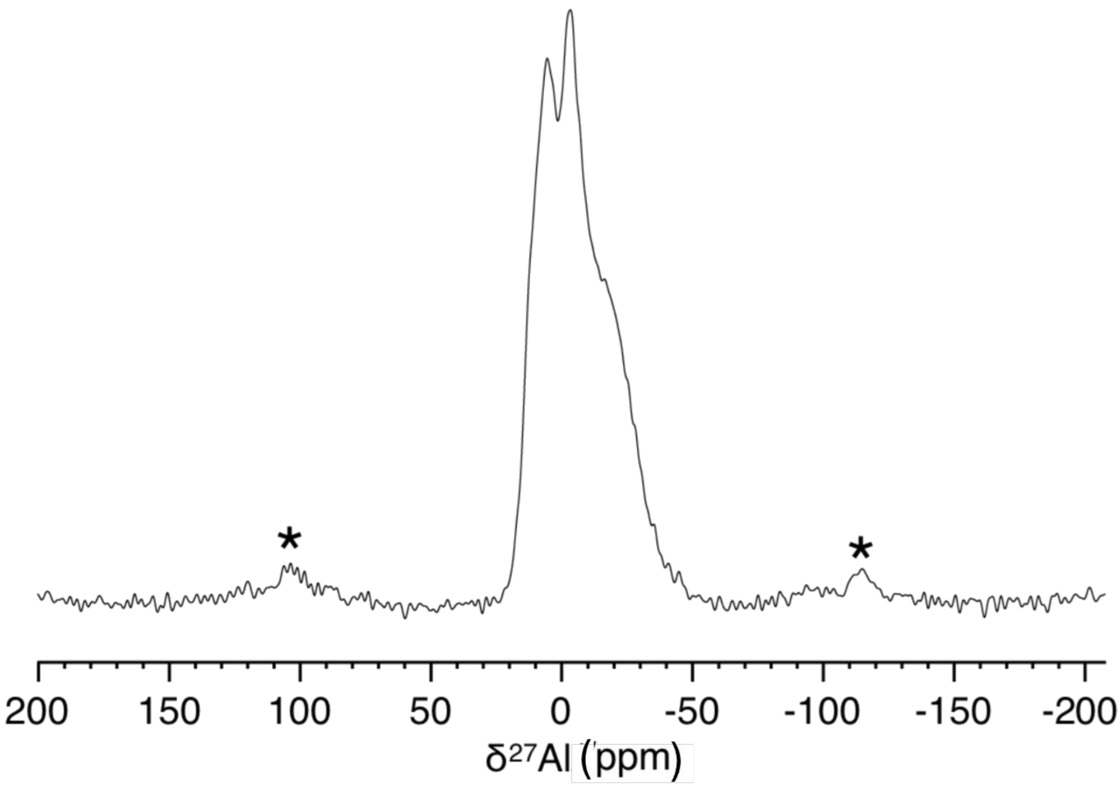

Figure S10. 1D ^{27}Al Hahn-echo MAS spectrum of ^{27}Al of **Al-L-Asp_ww** acquired at 16.4 T (701 MHz of proton Larmor frequency), using 3.2 mm rotor spinning at 20 kHz of MAS frequency and 270 K of probe incoming air temperature. Asterisks denote spinning sidebands.

Table S2. ^{27}Al isotropic chemical shift ($^{iso}\delta^{27}Al$), quadrupolar coupling constants (C_Q), asymmetry parameter (η_Q), line broadening window function (FWHM) and relative site abundance extrapolated from the fitting of 1D ^{27}Al Direct Excitation MAS (zg) MAS spectrum Al-L-Asp, Fig 3), under the hypothesis that two aluminum species are present within the material.

	$^{iso}\delta_{27Al}$ (ppm)	C_Q (MHz)	η_Q	Gaussian FWHM (Hz)	Abundance (%)
**Al1**	17.15 ± 0.03	11.980 ± 0.005	0.378 ± 0.002	(235 ± 1)·10	68.45 ± 0.06
**Al2**	17.12 ± 0.01	9.090 ± 0.003	0.3686 ± 0.0006	678 ± 3	31.55 ± 0.06

4. Optimization of the activation procedure and textural properties

Table S3 CHN analysis on **Al-L-Asp_ww** and theoretical percentage

Element	Theoretical wt% Al(OH)(Aspartate)	Experimental wt%	Theoretical Wt% [Al(OH)(Aspartate)(HNO_3)$_{0.31}$] 1.5 (H_2O)
C	27.11	21.44	21.5
H	3.9	4.15	4.16
N	7.9	8.57	8.5

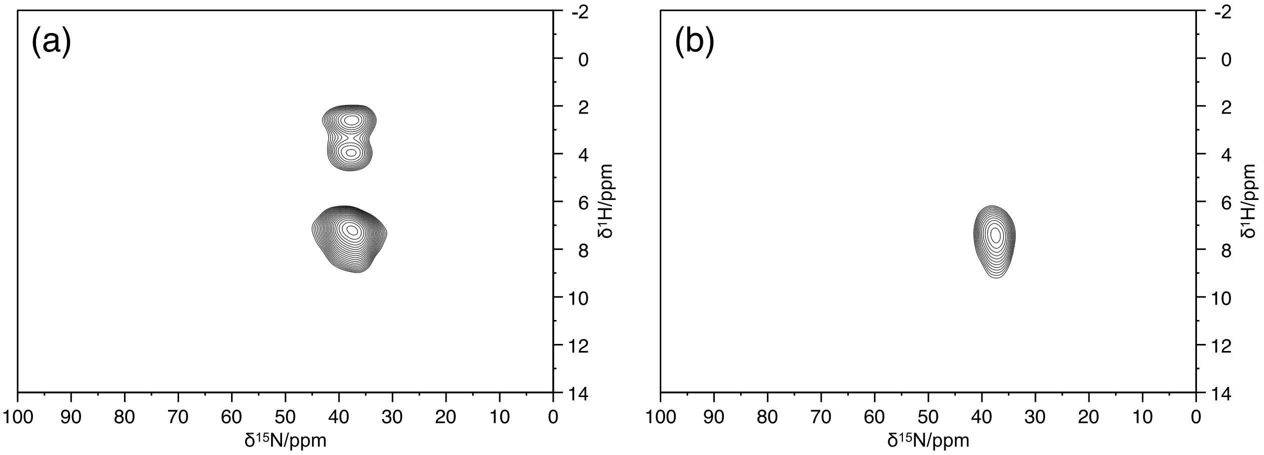

Figure S11. 2D ^{1}H-^{15}N FSLG HETCOR spectra of ^{15}N labelled [**Al-L-Asp_200**] acquired with two different contact times: 1500 µs and 100 µs, respectively **(a)** and **(b)**. The spectra were acquired at 20.0 T (850 MHz of proton Larmor frequency), using 3.2 mm rotor spinning at 20 kHz of MAS frequency and 270 K of probe incoming air temperature.

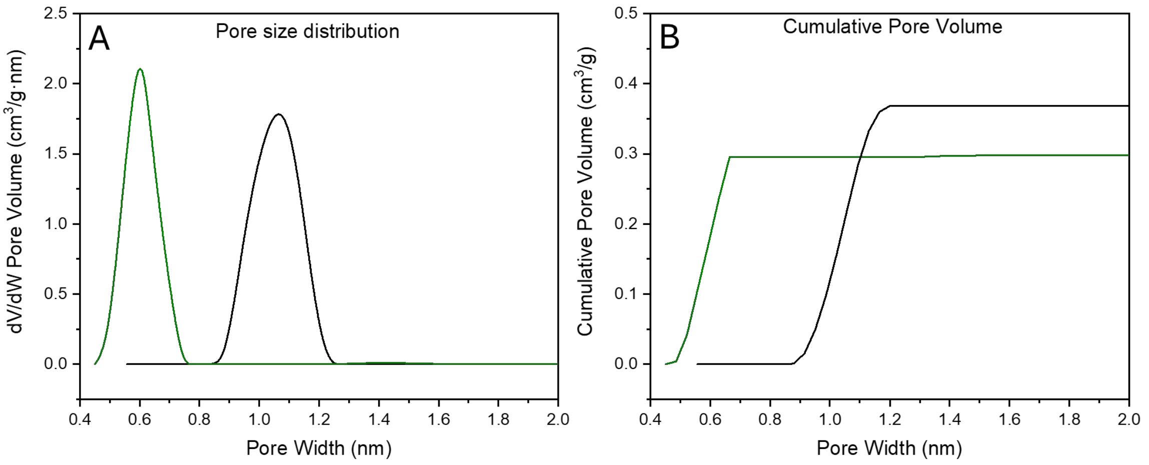

Figure S12. Pore size distribution calculated on N_2 adsorption curves of: **Al-L-Asp_200** (green curve) and Al-FUM (black curve) (**A**) and Cumulative Pore Volume of: **Al-L-Asp_200** (green curve) and Al-FUM (black curve) (**B**).

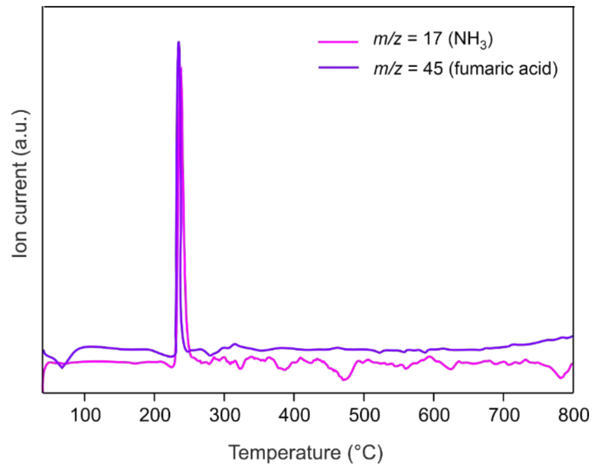

Figure S13. Mass peaks observed during the thermal decomposition of **Al-L-Asp_ww**.

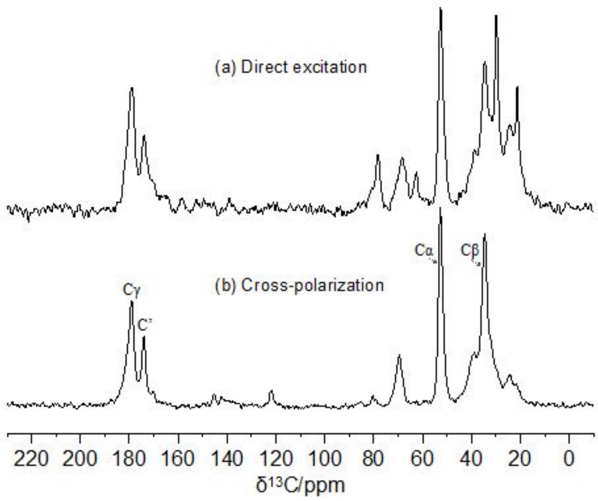

Fig S14. Comparison of 1D {^{1}H}-^{13}C CP MAS spectrum (b) acquired with a long contact time of 1000 μs, and 1D ^{13}C Hahn-echo MAS spectrum (a) of [**Al-L-Asp_ww**]. The spectra were acquired at 20.0 T (850 MHz of proton Larmor frequency) and -3 °C of probe incoming air temperature, using 3.2 mm rotor spinning at 20.0 kHz of MAS frequency and 80 kHz of ^{1}H decoupling. The spectra are normalized setting the same Cα peak height. Comparing the {^{1}H}-^{13}C CP MAS with the direct excitation (DE) ^{13}C Hahn-Echo MAS spectrum we see a few new signals at δ_{13C}= 80 ppm and δ_{13C}= 20-30 ppm that can be attributed to a little amount of free GVL remained trapped in the MOF pores. These peaks are weakly visible also in the CP spectrum, probably corresponding to little amount stably adsorbed on the MOF surface, not excluding that some of them could coordinate the MOF aluminum node. In the direct excitation spectrum are observable also flexible and liquid components that are too mobile to be observed in the CP spectrum.

5. CO_2 adsorption properties

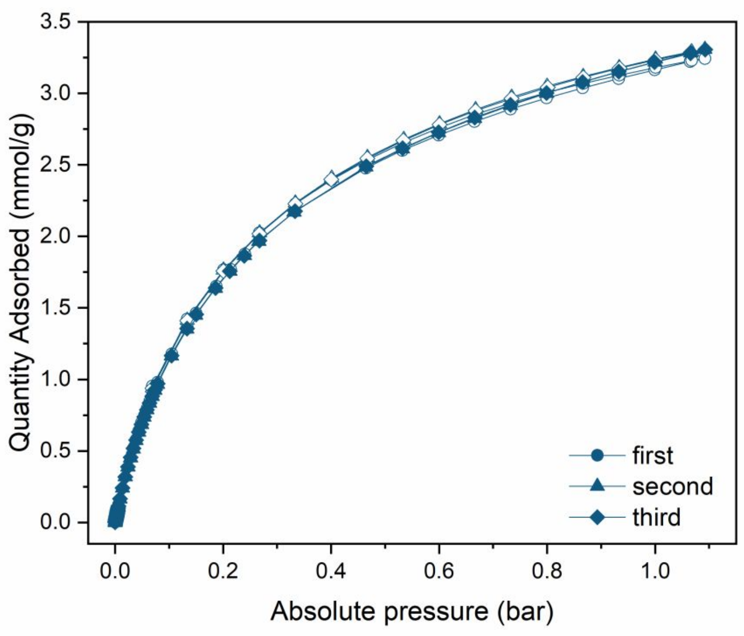

Fig S15. First (circles), second (triangle) and third (rhombus) cycles of CO_2 adsorption at 0°C on **Al-L-Asp_200.**

6. Tables of NMR Experimental Parameters

Table S4. Acquisition parameters for ^{1}H-^{13}C Cross-Polarization MAS (CP-MAS) and ^{1}H-^{13}C HETCOR at 20.0 T (850 MHz of ^{1}H Larmor frequency) spectrometer for [**Al- L-Asp_ww**], 3.2 mm probe

**Experimental Parameters**	^{1}H	^{13}C	
calibrated π/2 pulse (μs)	2.531	3.376	
	**CP-MAS**	**HETCOR**	
Acquired Nuclei	^{13}C	^{1}H	^{13}C
MAS Frequency (kHz)	20.0	20.0	
Number of scans	28672	352	
Recycle delay (s)	2.0	2.0	
Spectral width (kHz)	104.17	52.96	90.91
Acquisition time (ms)	14.95	1.89	13.66
^{1}H CP sequence	ramp 70% to 100%	ramp 70% to 100%	-
^{1}H CP power (100%) (kHz)	74.44	74.44	-
^{13}C CP sequence	constant power	-	constant power
^{13}C CP power (kHz)	54.23	-	54.23
^{1}H Decoupling sequence	SPINAL-64[a]	SPINAL-64[a]	-
^{1}H Decoupling power (kHz)	80.00	80.00	-
^{1}H homonuclear Power (FSLG)[b] (kHz)	-	100.00	-
Real acquired points	3114	200	2484
Window function	Exponential (LB: 100 Hz)	Square Sine Bell (SSB: 3)	Exponential (LB: 100 Hz)
Real processed points	32768	2048	4096

[a] Fung, B. M.; Khitrin, A. K.; Ermolaev, K. *J. Magn. Res.* **2000**, *142*, 97–101.

[b] Lee, M.; Goldburg, W.I. *Phys. Rev.* **1965**, *140*,1261-1271.

Table S5. Acquisition parameters for ^{13}C 1D Hahn-echo with ^{1}H decoupling at 20.0 T (850 MHz of ^{1}H Larmor frequency) spectrometer for [**Al-L-Asp_ww**], 3.2 mm probe.

**Hahn Echo Experimental Parameters**	^{1}H	^{13}C
calibrated π/2 pulse (μs)	2.531	3.376
Acquired Nucleus	^{13}C	
MAS Frequency (kHz)	20.0	
Number of scans	43792	
Recycle delay (s)	15.0	
Spectral width (kHz)	104.17	
Acquisition time (ms)	14.95	
^{1}H Decoupling sequence	SPINAL-64[a]	
^{1}H Decoupling power (kHz)	80.00	
Real acquired points	3114	
Window function	Exponential (LB: 100 Hz)	
Real processed points	32768	

[a] Fung, B. M.; Khitrin, A. K.; Ermolaev, K. *J. Magn. Res.* **2000**, *142*, 97–101.

Table S6. Acquisition parameters for ^{27}Al 1D Direct Excitation MAS (zg) at 16.4 T spectrometer (701 MHz of ^{1}H Larmor frequency) for [**Al- L-Asp_ww**], 3.2 mm probe, Figure 3 main text.

**Direct Excitation Experimental Parameters**	^{27}Al
calibrated π/2 pulse (μs)	2.00
Acquired Nucleus	^{27}Al
MAS Frequency (kHz)	20.0
Number of scans	64
Recycle delay (s)	2.0
Spectral width (kHz)	133.90
Acquisition time (ms)	3.34
Real acquired points	500
Window function	Exponential (LB: 10 Hz)
Real processed points	65536

Table S7. Acquisition parameters for ^{1}H-^{15}N Cross-Polarization MAS (CP-MAS) at 20.0 T spectrometer (850 MHz of ^{1}H Larmor frequency) for [**Al-** L**-Asp_ww**], 3.2 mm probe.

**Experimental Parameters**	^{1}H	^{15}N		
calibrated π/2 pulse (μs)	2.531	7.778		
	**CP-MAS**	**HETCOR**		
Acquired Nuclei	^{15}N	^{1}H		^{15}N
MAS Frequency (kHz)	20.0	20.0		
Number of scans	92464	80*		192**
Recycle delay (s)	3.0	2.8*		2.0**
Spectral width (kHz)	50.00	52.96		20.00
Acquisition time (ms)	14.94	0.94*	2.42**	14.90
^{1}H CP sequence	ramp 70% to 100%	ramp 70% to 100%		-
^{1}H CP power (100%) (kHz)	53.96	53.96		-
^{15}N CP sequence	constant power	-		constant power
^{15}N CP power (kHz)	30.59	-		30.59
^{1}H Decoupling sequence	SPINAL-64[a]	SPINAL-64[a]		-
^{1}H Decoupling power (kHz)	88.00	88.00		-
^{1}H homonuclear Power (FSLG)[b] (kHz)	-	100.00		-
Real acquired points	1494	100*	256**	596
Window function	Exponential (LB: 50 Hz)	Square Sine Bell (SSB: 2)		Exponential (LB: 100 Hz)
Real processed points	32768	2048		4096

* CP Contact time = 50 μs.

** CP Contact time = 1000 μs.

[a] Fung, B. M.; Khitrin, A. K.; Ermolaev, K. *J. Magn. Res.* **2000**, *142*, 97–101.

[b] Lee, M.; Goldburg, W.I. *Phys. Rev.* **1965**, *140*,1261-1271.

Table S8. Acquisition parameters for ^{15}N 1D Direct excitation at 20.0 T spectrometer (850 MHz of ^{1}H Larmor frequency) for [**Al- L-Asp_ww**], 3.2 mm probe, Figure 5 main text.

**Experimental Parameters**	^{1}H	^{15}N
calibrated π/2 pulse (μs)	2.531	7.778
Acquired Nucleus	^{15}N	
MAS Frequency (kHz)	20.0	
Number of scans	81788	
Recycle delay (s)	4.0	
Spectral width (kHz)	50.00	
Acquisition time (ms)	14.94	
^{1}H Decoupling sequence	SPINAL-64[a]	
^{1}H Decoupling power (kHz)	88.00	
Real acquired points	1494	
Window function	Exponential (LB: 50 Hz)	
Real processed points	32768	

[a] Fung, B. M.; Khitrin, A. K.; Ermolaev, K. *J. Magn. Res.* **2000**, *142*, 97–101.

Table S9. Acquisition parameters for 1D ^1H spectrum acquired at 16.47 T (700 MHz of ^1H Larmor frequency) spectrometer for the [**Al-L-Asp_200**] digested in D_2O sample of Figure S12.

**Direct Excitation with H_2O Presaturation Experimental Parameters**	^1H
Calibrated π/2 pulse (μs)	11.55
Number of scans	1024
Recycle delay (s)	10.0
Spectral width (kHz)	20.00
Acquisition time (s)	1.63840
H_2O presaturation power (Hz)	0.0707
Real acquired points	65536
Window function	Exponential (LB: 0.30 Hz)
Real processed points	262144

Table S10. Acquisition parameters for ^{27}Al 2D MQMAS (3QMAS) with z-filter at 16.4 T spectrometer (701 MHz of ^{1}H and 182.70 MHz for ^{27}Al Larmor frequencies) for [**Al- L -Asp_ww**], 3.2 mm probe, Figure 3 main text.

**Experimental Parameters 3QMAS with z-filter**	^{27}Al	
calibrated π/2 pulse low-power regime	10.00 μs @ 9.2 W	
Acquired Nuclei	^{27}Al	^{27}Al
MAS Frequency (kHz)	20.0	
Number of scans	2048	
Recycle delay (s)	1.0	
Spectral width (kHz)	74.63	20.00
Acquisition time (ms)	13.72	1.18
Excitation pulse	9.70 μs @ 300 W	
Conversion pulse	2.40 μs @ 300 W	
Selective excitation pulse	10.00 μs @ 9.2 W	
z-filter delay (μs)	20	
Real acquired points	2048	46
Window function	Exponential (LB: 150 Hz)	Exponential (LB: 100 Hz)
Real processed points	16384	1024

7. Solution NMR Analysis of the digested MOF Al-L-Asp

A sample of freshly re-prepared Al-L-Asp was cleaned washing it in hot deionized water at T = 70 °C for 2 h and then dried overnight in a static oven at T = 120°C as reported in the experimental part. One part of the sample was activated by using a high-vacuum glass line (residual pressure P < 10^{-4} mbar) and warming it at T = 200 °C overnight. Figure S7 reports the solid-state CP MAS spectra of the reprepared and washed sample [**Al-L-Asp_ww**] (black line), overlapped with the same sample after the activation treatment [**Al- L -Asp_ww**] (red line). The spectrum of [**Al- L -Asp_ww**] is analogous to the spectrum reported in Figure 2 (main text) on the previously prepared (but analogous) sample [**Al-L-Asp**]. The activated sample, from one side, shows the loss of some resonance of volatile impurities δ_C ~75 ppm and δ_C = 20-30 ppm that can be assigned to residual GVL not completely removed during the washing step. From the other side, it shows the appearance of strong resonances at δ_C =140 ppm and δ_C = 170 ppm that can be attributed to the formation of significant amounts of fumarate, by elimination of NH_3 from the aspartate linker. A small signal at about δ_C = 70 ppm can be attributed to the formation of malate as previously observed in [**Al- L - Asp**].

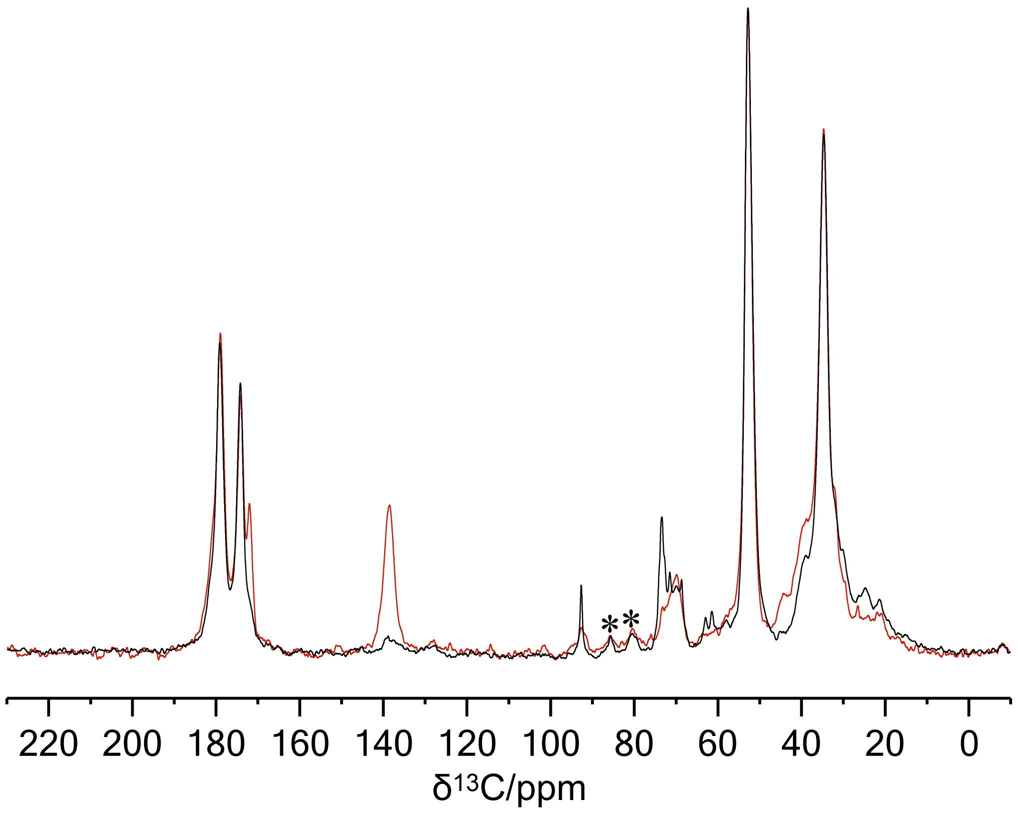

Fig. S16. {^{1}H}-[13]C Cross-Polarization (CP MAS) spectra comparison of the reprepared washed Al-Asp [**Al-L-Asp_ww**] (**black**), and [**Al-L-Asp_200**] (**red**). The spectra were

acquired with a long contact time of 1000 μs, at 20.0 T (850 MHz of proton Larmor frequency) and T = -3 °C of probe incoming air temperature, using 3.2 mm rotor spinning at 20.0 kHz of MAS frequency and 80 kHz of ^{1}H decoupling. Asterisks denoted spinning sidebands. The spectra are normalized setting the same Cα peak height.

A small portion of the activated sample (6.5 mg) was solubilized in D_2O through addition of an excess of hydrofluoric acid 40% (HF); the sample was then analyzed by solution NMR. Figure S8 reports the solution NMR spectrum with the assignment of the most significant species. Notably, we found a significant amount of fumaric acid, as observed in the ssNMR spectrum above, that can be quantified from the resonance integrals as the 30 % of the amount of aspartic acid. Assuming that the fumaric acid is entirely produced during the activation step, we can calculate that 23% of the initial aspartate is degraded during the activation step.

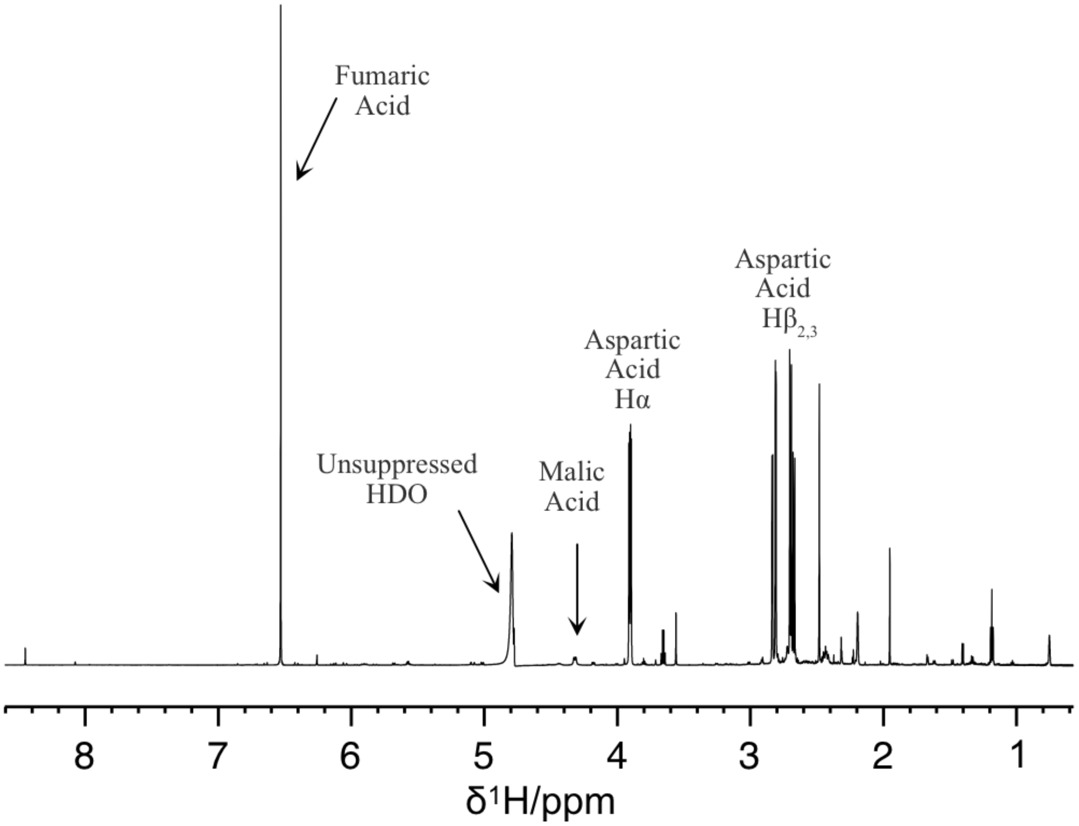

Fig. S17. 1D ^{1}H spectrum of the dissolved MOF **Al-L-Asp_200** in D_2O 16.47 T (700 MHz of proton Larmor frequency) and T= 25 °C with a water presaturation of 0.07 Hz. On the spectrum the assignment of the more significant compounds found are reported. Chemical shifts are referred to TSP standard.

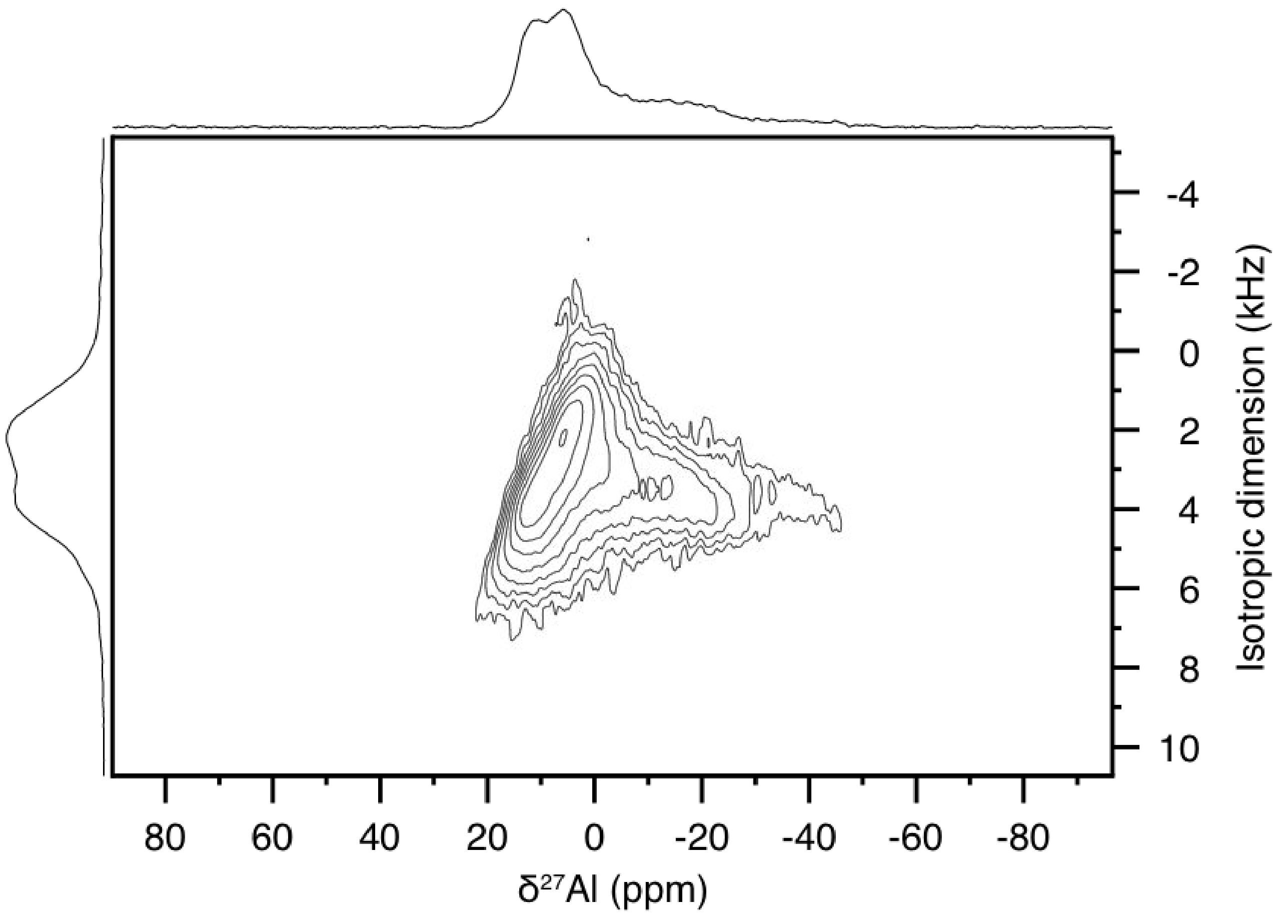

Figure S18. 2D ^{27}Al MQMAS (3QMAS) spectrum of **Al-L-Asp_ww** acquired at 16.4 T (701 MHz of proton Larmor frequency, 182.7 MHz of ^{27}Al Larmor frequency), using 3.2 mm rotor spinning at 20 kHz of MAS frequency and 270 K of probe incoming air temperature. In the indirect Isotropic dimension, we can clearly distinguish two components, also clarified in the projection spectrum on the left side of the spectrum.
